# Targeting Post‐Irradiation Thyroid Dysfunction: Electrospun Scaffolds As A Dual‐Action Approach for Antioxidant and Immune Modulation

**DOI:** 10.1002/adhm.202501857

**Published:** 2026-02-03

**Authors:** Maria Heim, Ella‐Louise Handley, Daniel Grant, Lizi M. Hegarty, Elaine Emmerson, Anthony Callanan

**Affiliations:** ^1^ Institute for Bioengineering, School of Engineering University of Edinburgh Edinburgh UK; ^2^ Centre for Regenerative Medicine, Institute for Regeneration and Repair University of Edinburgh Edinburgh UK; ^3^ Centre for Inflammation Research, Institute for Regeneration and Repair University of Edinburgh Edinburgh UK

**Keywords:** adenosine, electrospinning, immunomodulation, macrophages, radiation injury, thyroid, tissue engineering

## Abstract

Radiation‐induced hypothyroidism (RIHT) is a frequent consequence of head and neck radiotherapy, driven by oxidative stress, inflammation, and immune dysregulation. Current therapies address hormonal imbalance but not underlying tissue damage. Strategies involving macrophage modulation and oxidative stress reduction represent a promising target for restoring homeostasis in the irradiated thyroid. Electrospun polycaprolactone (PCL) scaffolds incorporating 0.5%–3% adenosine are developed to provide localized modulation of oxidative and inflammatory responses. Adenosine incorporation does not alter scaffold morphology or stability. In vitro studies demonstrate that 1% adenosine scaffolds enhance thyrocyte proliferation, epithelial cohesion, and expression of antioxidant enzymes glutathione peroxidase (*GPX1*) and catalase (*CAT*), while reducing markers of senescence and apoptosis (*RGN*, *CDKN2A*, *CASP3*). In parallel, adenosine scaffolds regulate THP‐1‐derived macrophage behaviour, promoting a pro‐reparative CD206+/CD163+ phenotype and reducing CD86, *CD80*, and *TNF*α expression associated with inflammatory activation. This study identifies fibrosis and oxidative stress as contributors to RIHT and demonstrates the feasibility of adenosine‐blended scaffolds as a platform for targeting these mechanisms. Macrophage heterogeneity was characterized in the thyroid pre‐ and post‐irradiation for an immune‐guided design. The resulting scaffolds provide a targeted strategy to modulate key contributors to RIHT pathology, laying the groundwork for future in vivo validation.

## Graphical abstract

Radiation‐induced hypothyroidism follows head and neck radiotherapy due to oxidative stress and inflammation. Electrospun polycaprolactone scaffolds containing adenosine have potential to modulate thyroid repair. Scaffolds enhance thyrocyte proliferation, antioxidant enzymes glutathione peroxidase and catalase, reduce senescence and apoptosis markers, and shift macrophages toward a pro‐reparative phenotype, supporting immune‐guided biomaterial therapy after irradiation and inflammatory dysregulation in thyroid tissue.

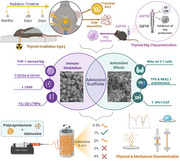

## 1 Introduction

Radiation‐induced hypothyroidism (RIHT) affects approximately 92% of head and neck cancer patients and up to 20% of breast cancer patients post‐radiotherapy [1, 2]. Signified by the reduction of thyroid hormone triiodothyronine (T3) and thyroxine (T4) levels, the disease significantly reduces the quality of life of the afflicted [3]. Patients experience a vast range of symptoms, affecting but not limited to the cardiovascular, neurological, metabolic, and gastrointestinal systems. The only current treatment option, hormone replacement therapy, comes in the form of synthetic T4, but presents with several drawbacks. Patients report persisting symptoms and multiple drug interactions with an extensive list of medications and nutritional components [4]. Additionally, the incidence rate of RIHT is steadily rising due to an increase in head and neck cancer survivors, underscoring the need for novel therapeutics [5]. While treatments provide the greatest therapeutic effect when tailored to the type of injury and resulting tissue dysregulation, the aetiology of RIHT and effects of head and neck irradiation (IR) on the thyroid microenvironment remain to be elucidated.

Multiple mechanisms of tissue damage have been suggested to contribute to the decrease of hormone production found during RIHT. IR‐induced apoptosis of hormone‐producing thyrocytes, gland capsule fibrosis, atherosclerosis, thyroiditis, and the formation of thyroglobulin (Tg) autoantibodies are commonly indicated hypotheses [6, 7, 8, 9]. IR injury is generally divided into early‐ and late‐stage injury, with the first being marked by the production of radical oxygen species (ROS) and recruitment of inflammatory cells to the site of injury. This is followed by a period of latency and late‐phase injury, characterized by chronic inflammation and fibrosis onset [10, 11, 12]. We previously gathered data utilizing a murine model of thyroid radiation injury, which indicated extracellular matrix (ECM) deposition in the thyroid gland correlating with mechanisms of fibrosis, which are a common consequence of IR injury and are known to contribute to fibrosis [13, 14, 15, 16].

Macrophages (Mφ), a type of immune cell, have been widely recognized as key regulators of both fibrosis and tissue regeneration. However, their highly plastic and dynamic phenotype allows them to contribute to a range of reparative processes, including anti‐inflammatory signalling, ECM remodelling, and angiogenesis [17, 18]. In this context, modulation of Mφ presents an attractive therapeutic target due to their dual roles in promoting fibrotic pathology and mediating regenerative responses, which is already exploited for therapeutic benefits against other conditions [19, 20, 21]. Characterization of the Mφ compartment under homeostatic and IR conditions provides foundational insight into the immune landscape of the relevant tissue, enabling informed therapeutic design aimed at guiding these cells toward beneficial pro‐repair, anti‐fibrotic phenotypes. Recently, such therapeutic approaches have included the use of tissue‐engineered electrospun scaffolds to influence Mφ plasticity [22, 23, 24].

Electrospun scaffolds are 3D ECM‐mimicking platforms which provide great promise for endocrine tissue regeneration [25, 26]. Compared to other fabrication techniques, such as hydrogels or solvent‐cast films, electrospinning allows precise control over fibre diameter and orientation, as well as composition, mechanical properties and topography, enabling fine‐tuning of the microenvironment to replicate physiological tissue architecture [26, 27, 28, 29, 30, 31]. This approach produces highly porous, fibrous matrices that closely mimic the extracellular structure of native thyroid tissue, supporting cell adhesion, nutrient diffusion, and 3D organisation [32, 33]. These characteristics make electrospun scaffolds well suited for studying thyroid cellular behaviour and microenvironmental responses [33, 34]. Furthermore, the properties of electrospun scaffolds provide a significant advantage in the delivery of drugs and bioactive molecules for local environment modulation [35, 36, 37, 38]. Recent biomaterials research has demonstrated that modifications in fibre architecture, surface chemistry, and composite design can be leveraged to regulate immune cell behaviour and enhance tissue regeneration [39, 40, 41].

Polycaprolactone (PCL) is a biocompatible, biodegradable and non‐toxic polymer commonly employed within soft tissue engineering [42, 43, 44]. Various organic compounds have been integrated into electrospun fibres to influence Mφ plasticity, namely cytokines such as IL‐10 [45, 46], IL‐4 [47] and mechano growth factor (a splice variant of IGF‐1) [48], alongside biosynthesised compounds including polyphenols [49, 50], itaconic acid [51] and asiaticoside [52]. Although thyroid tissue engineering is largely still in its infancy, our previous work demonstrated the suitability of PCL scaffolds with a range of fibre diameters for the attachment and proliferation of primary and immortalized thyroid cells [26].

Several bioactive molecules show great potential for the fabrication of composite therapeutic scaffolds against RIHT, such as adenosine, which elicits a dual effect. Adenosine has been shown to promote Mφ class switching to a phenotype conducive for tissue regenerative effects. [53, 54]. Moreover, adenosine can play a cytoprotective role through increasing the expression of antioxidant enzymes such as superoxide dismutase, catalase (CAT), and glutathione peroxidase (GPX1) [55, 56]. This could increase clearance of IR‐induced ROS, reducing early‐stage injury. Blending adenosine within electrospun scaffolds provides the advantage of targeted delivery to the damaged or diseased tissue, overcoming the issues associated with intravenous administration due to its short half‐life and adverse effects on other systems, including the cardiovascular and skeletal systems [57]. Such work has been pursued for the promotion of osteogenesis in large bone defects [57] and enhanced analgesia during acupoint catgut embedding, a type of acupuncture [58]. Similar approaches have been explored for bioactive molecule incorporation in composite and hybrid scaffolds to improve biocompatibility and immunomodulatory outcomes across multiple tissue types [59, 60, 61].

This study aimed to develop a mechanistically informed scaffold platform to address key features of IR‐associated thyroid injury, specifically oxidative stress and ECM deposition. Adenosine was incorporated into electrospun PCL scaffolds for its antioxidant and immunomodulatory properties, which target these damage‐associated pathways. To guide scaffold design, thyroid Mφ heterogeneity was characterized under homeostatic and IR conditions, providing insight into immune cell populations relevant to biomaterial interactions. Complementary in vitro studies with IR Nthy‐ori 3‐1 thyroid and THP‐1‐derived Mφ cells further demonstrate the scaffold's relevance to IR‐induced tissue damage. This work represents the first application of adenosine‐blended electrospun PCL scaffolds for modelling and modulating thyroid cell–matrix interactions, introducing a novel biomaterial platform that combines targeted bioactivity with tunable structural design to explore thyroid repair and immune modulation following IR.

## 2 Results

### 2.1 Homeostatic Dysregulation in the Thyroid Following IR Injury

**FIGURE 1:**
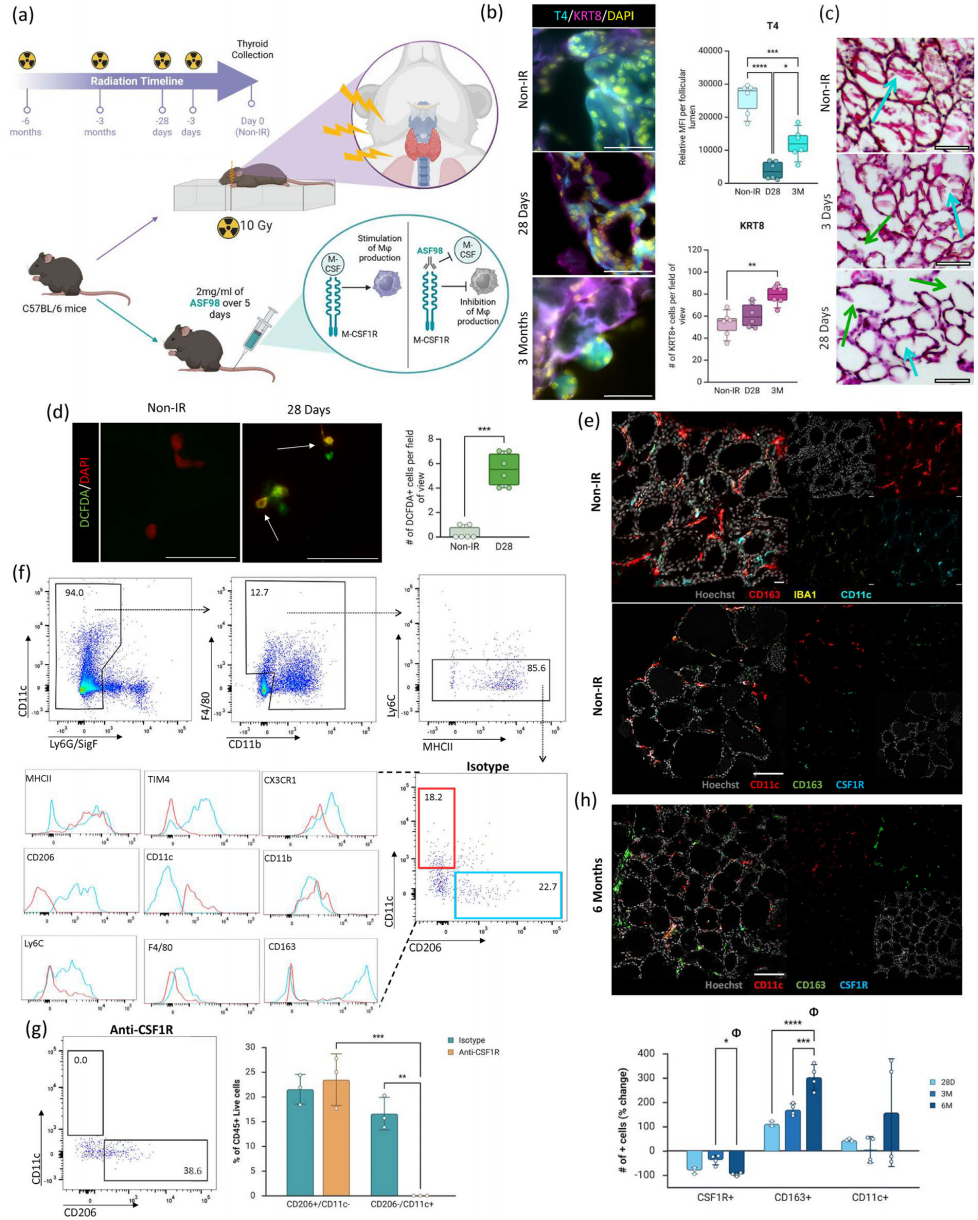
(a) Illustration of the irradiation (IR) timeline and anti‐CSF1R treatment (b) IF staining of thyroxine (T4, cyan) and cytokeratin 8 (KRT8, magenta) (N = 6) (c) H&E staining of thyroids pre‐ and post‐IR (Non‐IR, 3 Days, 28 Days). Blue arrows indicate the colloid, and green arrows follicle rupture. (d) Reactive oxygen species (ROS) quantification in thyroid tissue, pre‐ and 28 days post‐IR (N = 6). Statistical analysis: t‐test. (e) Representative IF images of thyroid tissue sections stained for CD163 (red), IBA1 (yellow) and CD11c (cyan) (top) as well as CD11c (red), CD163 (green) and CSF1R (cyan). (f) Gating strategy for identification of macrophage subpopulation in the healthy thyroid gland and representative expression of MHCII, TIM4, CD11c, CD11c, CD206, CX3CR1, Ly6C, F4/80, and CD163 (right). (N = 3) (g) Representative frequency of CD206+/CD11c‐ and CD206‐/CD11c+ macrophages in thyroids of mice administered anti‐CSF1R. (N = 3) (h) IF images of thyroid sections stained for CD11c (red), CD163 (green) and CSF1R (cyan) 6 months post‐IR. Quantification of myeloid populations in the thyroid post‐IR. (N ≥ 3) Φ = statistical significance compared to non‐IR. Statistical analysis: two‐way ANOVA and post hoc Tukey. Significance: *p ≤ 0.05, **p ≤ 0.01, ***p ≤ 0.001, ****p ≤ 0.0001. Boxplot = interquartile range and median, whiskers = 5th–95th percentile. Bar chart = mean ± SD. Scale bars = 60 µm, ×60 magnification.

To investigate the impact of IR on thyroid tissue, a mouse model of IR injury was utilized, as depicted in Figure 1a. Samples were harvested at multiple time points post‐IR: 28 days and 3 months for thyroid hormone and fibrotic deposition analysis, 3 and 28 days for histological analyses, 28 days for ROS quantification, 28 days, 3 months and 6 months for Mφ analyses. Non‐IR mice served as the baseline control group. These timepoints were selected to capture the progression of IR‐induced thyroid injury through early, intermediate, and chronic phases, reflecting both acute inflammatory and long‐term remodelling processes, as described previously [62, 63, 64]. ROS were not assessed at later stages as they are highly transient mediators acting predominantly during the acute post‐IR period [65, 66].

Confirmation of RIHT was obtained through immunofluorescence (IF) imaging of thyroid sections, revealing a marked reduction in T4 expression at both 28 days and 3 months post‐IR (Figure 1b and Figure S1a). Notably, post‐IR follicles displayed an increased presence of cytokeratin 8 (KRT8), a scaffolding protein associated with cellular stress responses and fibrosis. This upregulation was prominent around follicles lacking T4 at 3 months post‐IR, suggesting a contribution toward a non‐functional follicular phenotype. Histological evaluation using H&E, depicted in Figure 1c, revealed normal follicular morphology and colloid content (blue arrow) in non‐IR thyroids. In contrast, a progressive loss of colloid was observed in IR‐treated mice from day 3 to day 28, coinciding with increasing follicular rupture (green arrows). These structural changes support the onset and progression of RIHT following IR.

To evaluate oxidative stress as a potential mediator of tissue damage, intracellular ROS levels were quantified using DCFDA staining in primary thyroid cells isolated at 28 days post‐IR (Figure 1d and Figure S1b). Results demonstrated a significant increase in ROS accumulation relative to controls. Notably, ROS localization was predominantly nuclear, evidenced by the yellow color‐shift (white arrows) due to the overlay of DCFDA (green) and DAPI (red) signals, indicating increased oxidative stress.

In parallel, to characterize the Mφ compartment of the thyroid, immunofluorescent staining was performed (Figure 1e,f). Non‐IR thyroid tissue, as presented in Figure 1e, shows primarily distinct spatial expression of CD163, CSF1R, and CD11c within resident myeloid cells, while Figure 1f displays distinct expression of CD163, IBA1, and CD11c. These results demonstrate that thyroid‐resident Mφ in healthy mice comprise heterogeneous populations with overlapping expression of both pan‐Mφ markers (IBA1) and antigen‐presenting markers (CD11c), as well as the scavenger receptor CD163 and the colony‐stimulating factor 1 receptor (CSF1R), previously been shown to be essential for Mφ development and/or maintenance [62, 67]. To further define and quantify these populations, flow cytometry was conducted (Figure 1g). Two distinct Mφ subsets were identified: CD206+ CD11c‐ (red) and CD206‐ CD11c+ (blue). Marker expression profiles (MHCII, TIM4, CD11c, CD11c, CD206, CX3CR1, Ly6C, F4/80, and CD163) were visualized by histograms, confirming phenotypic diversity within the thyroid Mφ pool. Notably, the CD206‐ CD11c+ subset appeared to lack TIM4, associated with apoptotic cell phagocytosis [68], and CD163 expression, in comparison to the CD206+ CD11c‐ subset [69].

Many tissue‐resident Mφ in other organs are known to be reliant on Mφ colony stimulating factor (M‐CSF):colony stimulating factor 1 receptor (CSF1R) signalling for their development and maintenance [62, 67]. Here, to assess the dependency of the identified thyroid Mφ subpopulations on CSF1R signalling, healthy mice were treated with anti‐CSF1R‐blocking antibody (AFS98). This led to a significant depletion in the CD206‐CD11c+ Mφ subset (Figure 1g), validating their dependence on CSF1R signalling in the steady state thyroid. Together, this data provides a comprehensive baseline of thyroid Mφ heterogeneity.

Lastly, following characterisation of the homeostatic Mφ compartment, the composition and spatial distribution of thyroid myeloid populations were investigated over 6 months following IR (Figure 1h and Figure S2). Quantitative IF analysis indicated no significant change in CD11c+ cells, but a gradual increase in CD163+ cells. Furthermore, a significant reduction in CSF1R+ cells was noted at 6 months post‐IR. This pattern suggests a transition in the myeloid landscape characterized by loss or reprogramming of resident populations and accumulation of phenotypically diverse or monocyte‐derived cells. While potential variability was observed between the sexes concerning the response of CD11c+ cells, the dataset did not permit meaningful analysis but may present an avenue for future work. Although inter‐sex variability in terms of total cell numbers was observed in CSF1R+ and CD163+ cells, the overall trend was consistent across the cohort following adjustment for total cell numbers. This indicates that IR induces sustained remodelling of thyroid myeloid architecture.

### 2.2 Scaffold Fabrication & Characterization

#### 2.2.1 Morphology and Composition

The tissue damage and ECM remodelling observed in the thyroid post‐IR highlights the need for strategies to promote repair and restore immune homeostasis. A potential avenue for this includes the utilization of biomaterials blended with bioactives. As such, adenosine was selected due to its immunomodulatory properties.

**FIGURE 2:**
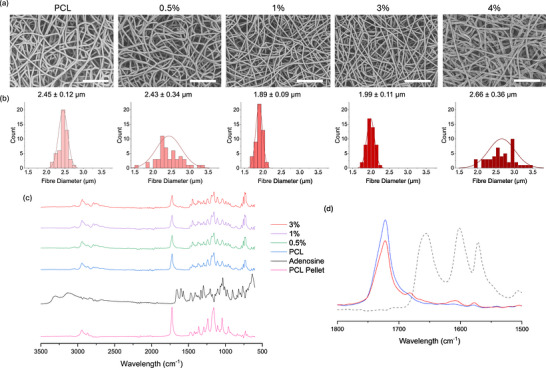
(a) SEM images of scaffolds at ×2000 magnification, (b) Fibre diameter distributions (N=50), (c) Full FTIR spectra of raw PCL and adenosine compared with the four scaffolds, and (d) overlaid FTIR spectra of the raw adenosine, PCL and 3% scaffold between 1500 and 1800 cm^−1^. Scale bars = 40 µm, ×1500 magnification.

Foremost, we investigated the maximum concentration of adenosine that can be blended within PCL scaffolds using needle electrospinning whilst conserving the desired morphology of randomly aligned, smooth and defect‐free fibres, with mean fibre diameters of approximately 2 µm [26]. Scanning electron microscopy (SEM) images of five scaffolds containing increasing concentrations of adenosine are displayed in Figure 2a. Scaffold groups are referred to via the % (w/v) adenosine concentration within the electrospinning polymer solution: PCL/0%, 0.5%, 1%, 3% and 4%. However, assuming complete solvent evaporation and no degradation of materials during the electrospinning process, the actual adenosine content within the dry fibres are 0%, 5%, 10%, 30%, and 40% (w/w) relative to PCL. These values are derived by comparing the mass of adenosine to the mass of PCL within the electrospinning solution.

The SEM images demonstrate similar morphologies between the PCL, 0.5%, 1% and 3% scaffolds, whereby the fibres are randomly aligned and free of defects such as beads. The surfaces of the PCL control, 1% and 3% fibres appear to be smooth, whilst the 0.5% fibres exhibit a more wrinkled morphology, suggesting some minor instabilities in electrospinning with this concentration. In contrast, electrospinning with a 4% adenosine solution was highly unstable, producing flatter and discontinuous ribbon‐like structures. This suggests that the maximal limit of adenosine whilst maintaining PCL fibre integrity is approximately 3%. As such, the 4% adenosine scaffold was excluded from further material and in vitro characterizations.

Figure 2b displays the fibre diameter distributions for the five scaffolds. Fibre diameters ranged between 1.89 and 2.66 µm for all scaffold groups. Incorporation of adenosine appears to decrease the fibre diameter with the exception of the 4% scaffold, likely due to the flattened structure of the fibres. The PCL, 1% and 3% scaffolds exhibited a relatively narrow distribution of the fibre diameters, indicative of a stable electrospinning process and the formation of a uniform network of fibres. However, a broader distribution of fibre diameters were observed with both the 0.5% and 4% adenosine scaffolds, whereby the unstable fibre formation resulted in more heterogenous morphologies.

Fourier transform infrared spectroscopy (FTIR) was performed to gather information on the functional groups present within the scaffolds. The FTIR spectra of the neat materials and electrospun mats in the range of 3500–500 cm^−1^ are presented in Figure 2c. The spectra of the bulk PCL and all scaffolds displayed the characteristic peaks for PCL, namely 2944 and 2866 cm^−1^ (C–H stretching: symmetrical and asymmetrical), 1724 cm^−1^ (C═O stretching), 1239 cm^−1^ (C‐O‐C asymmetrical stretching) and 1158 cm^−1^ (both C–O stretching and C–C stretching) [70, 71]. Similarly, neat adenosine showed expected bands including two broad peaks at 3304 and 3133 cm^−1^ (O‐H stretching attributed to the D‐ribose), 1035 cm^−1^ (C–O–H bending), and 1656 cm^−1^ (N–H bending), and mixed C═C and C═N stretching vibrations were at 1600 and 1571 cm^−1^ [72].

The characteristic peaks for adenosine are mostly obscured within the spectra of the adenosine fibres, which are dominated by the PCL absorption bands, likely due to its higher concentration. However, subtle peaks associated with N‐H bending and the mixed C═N and C═C stretching in the purine ring were observed within the 3% scaffold, indicating the presence of functional groups (2d). These bands demonstrated a blue shift toward higher wavelengths of 1681, 1608 and 1577 cm^−1^, respectively, arising from reduced hydrogen bond strength and electron transfer between the bonds likely due to drug‐matrix molecular interactions between the PCL and adenosine.

#### 2.2.2 Mechanical Characterization

**FIGURE 3:**
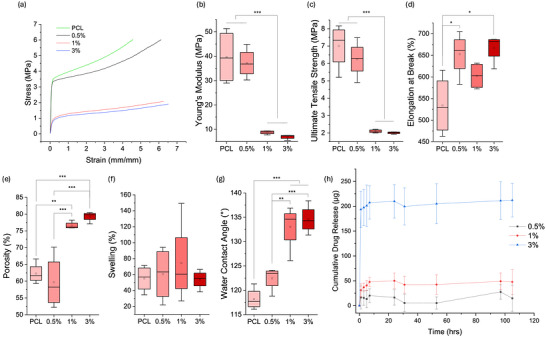
Mechanical and physical characteristics of the electrospun fibres. (a) Representative stress–strain curves under tension, (b) Young's Modulus, (c) Ultimate tensile strength, (d) Elongation at break, (e) Porosity, (f) Swelling after 48h incubation with phosphate buffered saline (PBS) at room temperature, (g) Water contact angle after 1s, (h) Cumulative release of adenosine. N = 4. Statistical analysis: two‐way ANOVA and post hoc Tukey, *p ≤ 0.05, **p ≤ 0.01, ***p ≤ 0.001. Boxplot: box = interquartile range and median, whiskers = 5th–95th percentile. Line graph: error bars = SD.

The mechanical properties of the scaffolds were measured using an Instron tensile testing machine. The average stress‐strain plots displayed in Figure 3a demonstrate the similar mechanical responses of the scaffolds under tensile load. All scaffolds followed the expected curves for a PCL electrospun graft, consisting of a linear elastic region followed by a singular yield point and a plastic region [73]. Principal differences were observed in scaffold mechanical properties, with both strength and stiffness decreasing with increased loading of adenosine. Looking at Young's modulus, Figure 3b, the PCL and 0.5% scaffolds overlap with the mean reported values of 39.70 and 37.17 MPa, respectively. Conversely, further increasing the adenosine concentration to 1% resulted in a 425% decrease in the Young's modulus. Whilst a decrease from 8.74 to 6.88 MPa was observed within the 1% and 3% scaffolds, respectively, this difference was not statistically significant.

Similarly, increasing the weight percentage of adenosine significantly reduced the maximal strength the material could withstand, correlating with the observed differences in Young's Modulus. Figure 3c indicates a threefold decrease of the ultimate tensile strength (UTS) when increasing the adenosine concentration from 0.5% to 1%. Although no statistical significance was observed between the UTS of the control and 0.5% scaffold, and the 1% and 3% scaffolds. In contrast, blending adenosine within PCL electrospun fibres appears to increase the ductile behaviour of the polymer. The elongation at break, Figure 3d, displayed an increasing trend with increasing loading of the nucleoside, with statistical significance reported between the control scaffold and both the 0.5% and 3% scaffolds.

#### 2.2.3 Physical Characterization & Release Kinetics

The porosity and degree of swelling of electrospun scaffolds influence cell infiltration and attachment, and the transport of nutrients and waste products [74]. Additionally, mechanical performance is correlated with the scaffold porosity, whilst the degree of swelling impacts the degradation profile and release kinetics of the adenosine. Greater porosity was observed in the scaffolds containing the highest concentrations of adenosine (Figure 3e). Interestingly, no differences in porosity were observed between the PCL and 0.5% group, or the 1% and 3% groups, suggesting that the structure of PCL/adenosine composite fibres is greatly affected between 0.5% and 1% loading. These values correlate with scaffold thicknesses, which increased from 38.8 ± 2.6 µm for the PCL control scaffold to 41.3 ± 10.3 µm, 62.3 ± 5.1 µm and 71.0 ± 4.7 µm for the 0.5%, 1% and 3% scaffolds, respectively. With regard to the degree of swelling, Figure 3f, no statistical difference was observed between all scaffold groups after 48h of incubation with phosphate buffered saline (PBS).

Scaffold hydrophilicity was analysed using the sessile drop method (Figure 3g). The water contact angle follows an increasing trend with increasing adenosine concentrations, signifying more hydrophobic behaviour. The 1% and 3% scaffolds demonstrated significance between both the PCL and 0.5% scaffolds, whilst again no significance was established between the PCL and 0.5% and the 1% and 3% scaffolds. Finally, the release of adenosine from the fibres into PBS at 37 °C was investigated, as demonstrated in Figure 3h. All scaffolds appear to show a burst release in the first 7 h, followed by a stable or increasing trend for up to 105h. Maximal adenosine concentration was relatively aligned with expected values, though the release from the 3% scaffold was approximately 4.5‐fold and 14‐fold greater than the 1% and 0.5% scaffolds, respectively, suggesting improved loading of adenosine at higher concentrations.

### 2.3 In Vitro Assessment of Electrospun Adenosine Scaffolds

#### 2.3.1 Response of Nthy‐Ori 3‐1 Cells

**FIGURE 4:**
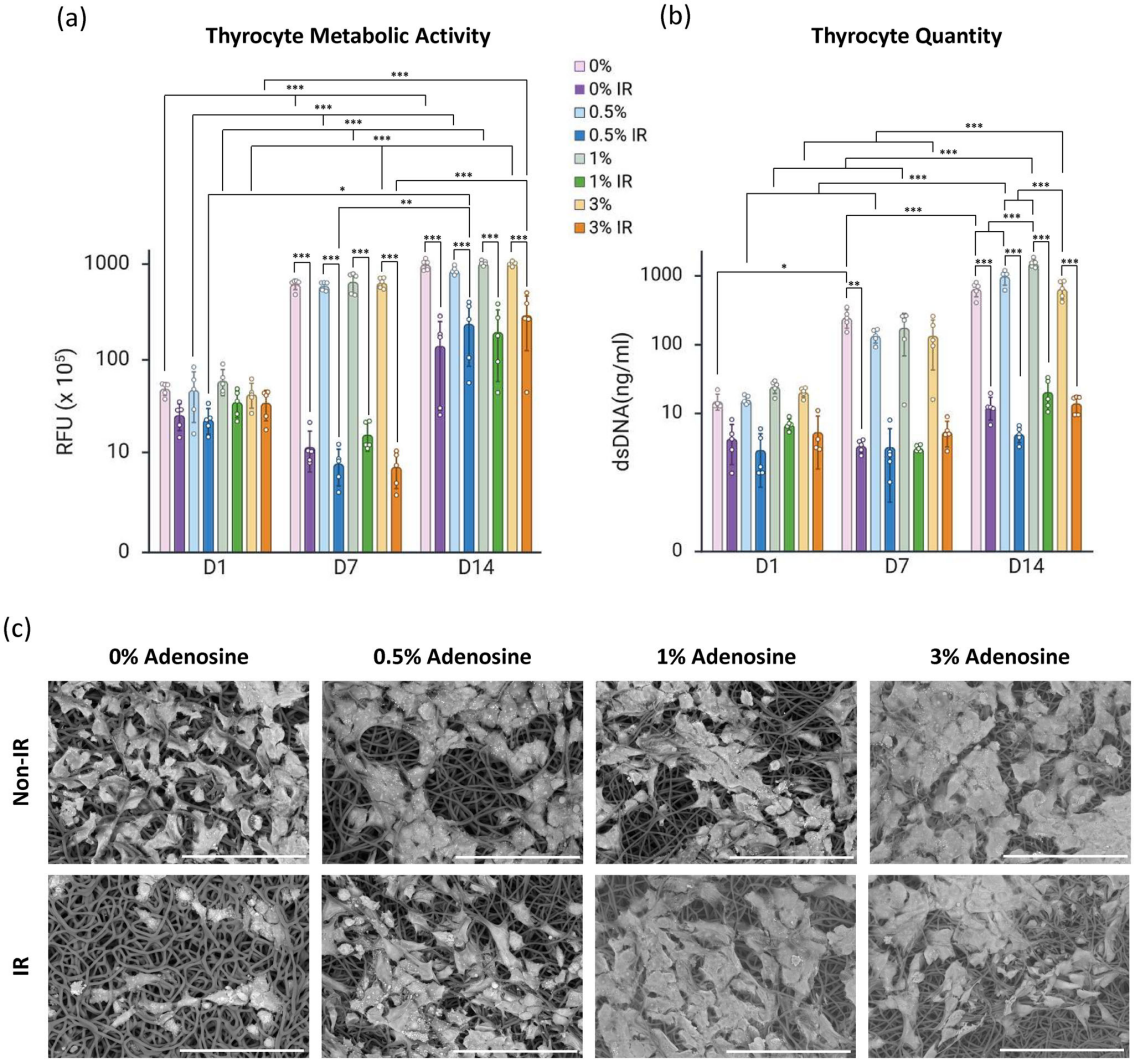
Responses of Nthy‐ori 3‐1 cells to electrospun adenosine scaffolds displaying: (a) metabolic activity measured via the CellTitre blue assay, (b) dsDNA quantification measured via the QuantIT PicoGreen assay and (c) representative osmium‐stained SEM at day 14. N = 5. Statistical analysis: two‐way ANOVA and post hoc Tukey, *p ≤ 0.05, **p ≤ 0.01, ***p ≤ 0.001. Data = mean ± SD. Scale bars = 100 µm.

To examine the influence of adenosine scaffolds on thyrocyte responses, Nthy‐ori 3‐1 cells were cultured on PCL‐only, 0.5%, 1%, and 3% adenosine scaffolds, with or without prior IR, and monitored over a 14‐day period. Metabolic activity progressively increased in all non‐IR groups throughout the study (Figure 4a), whereas IR‐treated cells exhibited a delayed response, with significant increases only between days 7 and 14. Across both time points, irradiated scaffolds displayed consistently lower metabolic activity than their corresponding non‐IR counterparts. Similarly, DNA content increased significantly in all non‐IR groups over time (Figure 4b). Although no differences were evident between non‐IR scaffolds at days 1 and 7, the 1% adenosine scaffold demonstrated a markedly higher DNA content than all other groups at day 14, indicating enhanced cell proliferation. In contrast, DNA content in irradiated scaffolds remained unchanged and was significantly reduced relative to the corresponding non‐IR groups at day 14. Notably, the PCL‐only scaffold was the only condition exhibiting a reduction in DNA content at day 7 following IR. Representative SEM images of osmium‐stained scaffolds revealed comparable cell morphology across all conditions, with no evidence of apoptotic features such as membrane blebbing (Figure 4c and Figure S4).

To determine whether the scaffold‐induced changes in metabolic activity were attributable to adenosine itself or required the biomaterial context, we additionally assessed the effects of free adenosine on non‐IR Nthy‐ori 3‐1 cells cultured on tissue‐culture plastic at concentrations equivalent to those found in scaffolds (Figure S3). No differences in metabolic activity were observed between groups containing 0%, 0.5% and 1% free adenosine, but overall metabolic activity increased in all these groups over time. In contrast, higher concentrations (3 and 4%) resulted in reduced metabolic activity, suggesting potential mild cytotoxic or inhibitory effects at these levels.

**FIGURE 5:**
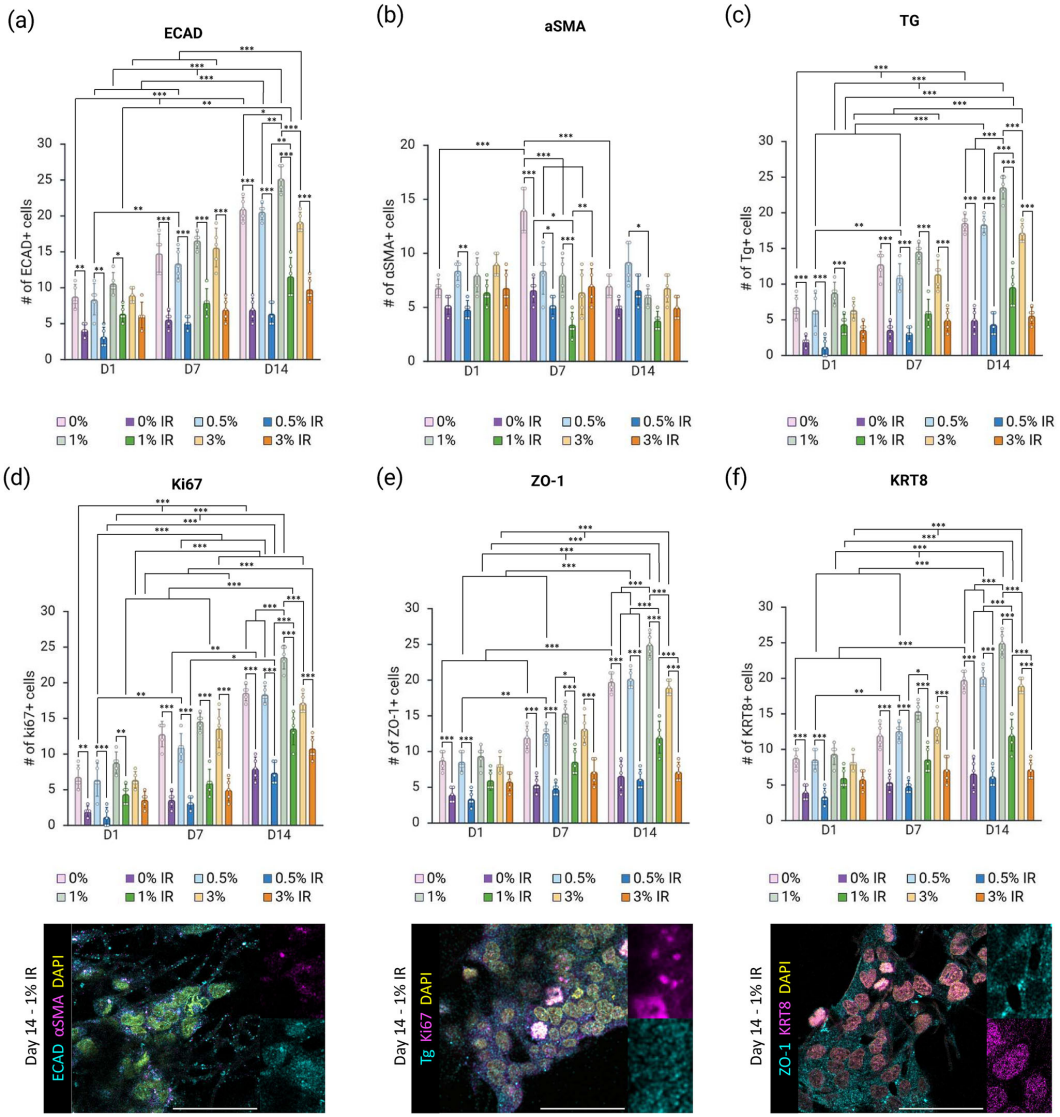
Responses of Nthy‐ori 3‐1 cells to electrospun adenosine scaffolds displaying: IF quantification of cell‐seeded scaffolds stained for (a) E‐Cadherin (ECAD) (b) α‐smooth‐muscle actin (αSMA), (c) Thyroglobulin (Tg), (d) Antigen Kiel 67 (Ki67) (e) Zonnula Occludens 1 (ZO‐1), and (f) cytokeratin 8 (KRT8). Representative images of 1% adenosine scaffolds seeded with irradiated cells is shown on the bottom. N = 5. Statistical analysis: two‐way ANOVA and post hoc Tukey, *p ≤ 0.05, **p ≤ 0.01, ***p ≤ 0.001. Data = mean ± SD. Scale bars = 60 µm.

IF analysis was conducted to assess epithelial integrity, thyroid‐specific functionality and ECM deposition across adenosine‐blended scaffolds. The number of ECAD+ cells increased steadily from day 1 to day 14 across all scaffold groups (Figure 5a). By day 14, the 1% adenosine scaffold supported the highest number of ECAD+ cells, showing significant differences compared to the PCL, 0.5%, and 3% groups. A similar trend was observed under IR conditions, where the 1% IR scaffold also exhibited higher ECAD+ cell numbers than the PCL‐only and 0.5% IR scaffolds. These findings indicate that moderate adenosine incorporation enhances epithelial cohesion and junctional organization, even following irradiation. In contrast, the number of αSMA+ cells, a marker associated with fibrotic remodelling and myofibroblast activation, peaked at day 7 in the PCL‐only group, where significantly higher levels were observed compared to all other conditions (Figure 5b). Interestingly, the 0.5% IR scaffold showed reduced αSMA+ expression at day 1 compared to its non‐IR counterpart, while the PCL‐only, 0.5%, and 1% IR groups each exhibited lower αSMA+ levels at day 7 relative to non‐IR scaffolds. Furthermore, fewer αSMA+ cells were detected on the 1% IR scaffold than on the PCL‐only scaffold at this timepoint. Together, these results suggest that the 1% adenosine scaffold supports a more favourable epithelial microenvironment, limiting fibrotic activation and promoting tissue homeostasis.

Quantification of Tg+ cells, a marker of thyroid‐specific function and pre‐courser of T4 and T3, showed a clear time‐dependent increase on all non‐IR scaffold groups, with the 1% condition supporting the highest number of Tg+ cells by day 14 compared to all other groups (Figure 5c and Figure S6). This indicates a clear advantage of this intermediate concentration for supporting thyroid‐specific function. In contrast, scaffolds seeded with IR cells exhibited markedly fewer Tg+ cells at all timepoints. Notably, the 1% IR scaffold was the only condition to demonstrate a progressive increase in Tg+ cells over the culture period, showing significantly higher levels than the PCL‐only and 0.5% IR scaffolds. Ki67+ cell quantification followed a similar trend. In non‐IR groups, the proportion of proliferating cells increased over time, with the 1% scaffold exhibiting the greatest rise by day 14 (Figure 5d and Figure S6). In contrast, scaffolds seeded with IR cells displayed a delayed proliferative response, with significant increases in Ki67+ cell numbers only between days 7 and 14. Across all timepoints, IR groups generally exhibited lower proliferation compared to their non‐IR counterparts, except for the 3% adenosine scaffold at day 1. Among IR conditions, the 1% scaffold again supported the highest number of Ki67+ cells, suggesting a partial preservation of proliferative capacity.

The number of ZO‐1+ cells increased progressively over time across all scaffolds seeded with non‐IR cells, indicating enhanced tight junction formation (Figure 5e and Figure S7). By day 14, the 1% adenosine scaffold supported the highest number of ZO‐1+ cells, showing significant increases compared to the PCL‐only, 0.5%, and 3% groups. A similar pattern was observed among IR scaffolds, where the 1% IR group displayed a greater number of ZO‐1+ cells at day 14 than other IR conditions, suggesting that moderate adenosine incorporation aids in the formation or maintenance of epithelial junctions even under IR stress. Nonetheless, all IR groups exhibited fewer ZO‐1+ cells than their non‐IR counterparts at days 7 and 14, with the PCL‐only and 0.5% IR scaffolds also displaying reduced ZO‐1 expression at day 1. KRT8+ cell counts followed a similar temporal pattern, increasing across all non‐IR groups over the 14‐day culture period (Figure 5f and Figure S7). The 1% adenosine scaffold consistently supported the greatest number of KRT8+ cells at both day 7 and day 14, consistent with improved maintenance of epithelial identity and cytoskeletal organisation. Following IR, this trend was partially preserved, with the 1% IR group exhibiting higher KRT8+ cell numbers than the 0.5% IR group at day 7, and all other IR scaffolds at day 14. Overall, all IR groups showed reduced KRT8+ cell counts compared to their corresponding non‐IR scaffolds at days 7 and 14, while only the PCL‐only and 0.5% IR groups were lower at day 1. Although the 3% scaffold also supported increased ZO‐1+ and KRT8+ expression, these effects were less pronounced than in the 1% group. Collectively, these results indicate that the 1% adenosine scaffold provides the most favourable microenvironment for preserving epithelial integrity and identity, both under homeostatic and IR conditions.

**FIGURE 6:**
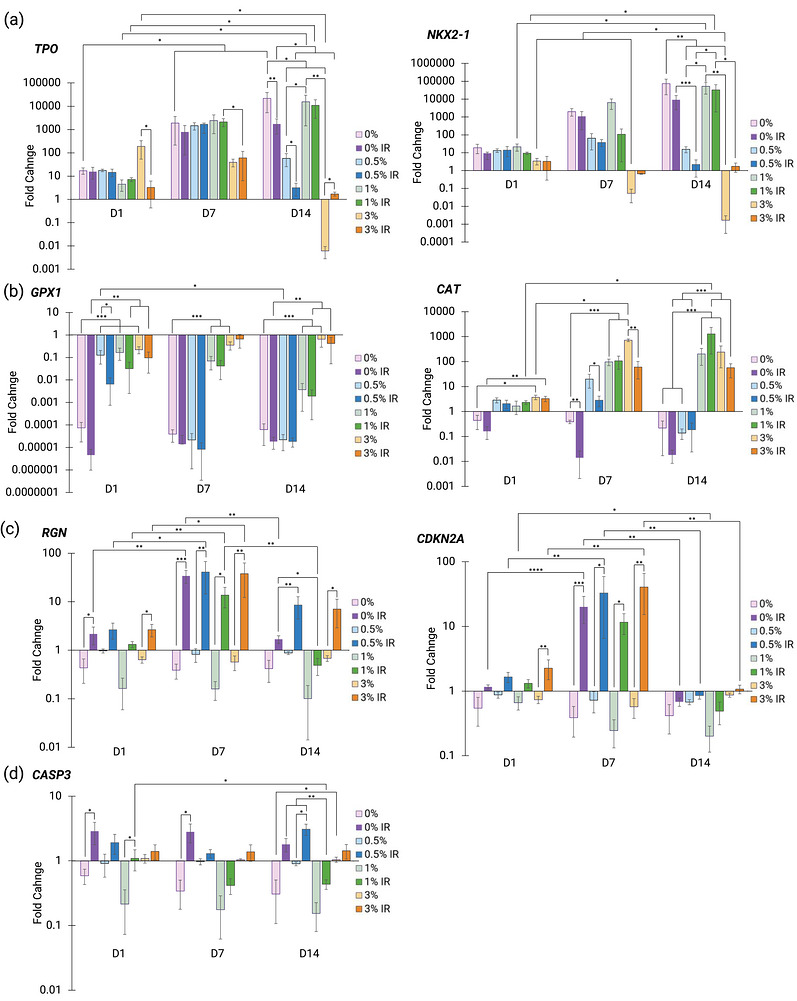
Responses of Nthy‐ori 3‐1 cells to electrospun adenosine scaffolds displaying: qRT‐PCR analysis of gene expression of (a) thyroid‐specific proteins thyroid peroxidase (*TPO*, left) and NK2 Homeobox 1 (*NKX2‐1*, right), (b) antioxidant enzymes glutathione (*GPX1*, left) and catalase (CAT, right), (c) senescence markers cyclin‐dependent kinase inhibitor 2A (*CDKN2A*, left) and regucalcin (*RGN*, right) as well as (d) the apoptosis‐associated protein caspase‐3 (*CASP3*). N = 5. Statistical analysis: two‐way ANOVA and post hoc Tukey, *p ≤ 0.05, **p ≤ 0.01, ***p ≤ 0.001. Data = mean ± SD.

Moreover, the relative gene expression of thyroid function–related proteins, antioxidant enzymes and markers of senescence as well as cell death were evaluated in reference to non‐IR Nthy‐ori 3‐1 cells cultured on tissue culture plastic, as shown in Figure 6a. Expression of thyroid peroxidase (*TPO*, left), a key enzyme involved in thyroid hormone synthesis, increased significantly from day 1 to day 14 in both the PCL‐only and 1% scaffold groups. Similarly, *TPO* expression rose in the 1% IR group over the study period, whereas all other IR conditions displayed reduced *TPO* expression at day 14 compared to their non‐IR counterparts. In contrast, *TPO* expression declined on the 3% scaffold over time and was significantly lower than both the PCL‐only and 1% groups at day 14. Although expression levels remained unchanged in the 0.5% and 0.5% IR groups, both were significantly lower than the respective PCL‐only controls at day 14. Expression of the thyroid transcription factor NK2 Homeobox 1 (*NKX2‐1*, right) followed a similar pattern, with upregulation in the PCL‐only and 1% groups and downregulation in the 3% condition. *NKX2‐1* expression was also significantly lower in the 0.5% group compared to the PCL‐only and 1% scaffolds at day 14. Among irradiated groups, the 0.5% and 3% IR conditions showed reduced *NKX2‐1* expression at day 14 relative to the PCL‐only and 1% IR groups, while the 1% IR scaffold exhibited a significant increase in expression over time. These findings suggest that moderate adenosine incorporation, as in the 1% scaffold, supports the maintenance of thyroid‐specific gene expression and functional activity, even following IR.

Expression of the antioxidant enzyme *GPX1*, shown in Figure 6b (left), was consistently higher in cells cultured on the 1% and 3% scaffolds compared to the PCL‐only group across all timepoints. This pattern was mirrored in the irradiated conditions, where the 1% and 3% IR scaffolds exhibited elevated *GPX1* expression relative to the PCL‐only IR group at every timepoint. In contrast, *GPX1* expression in cells on the 0.5% scaffold was initially higher at day 1 but declined significantly over the remainder of the study. Similarly, expression of the antioxidant enzyme *CAT* was significantly lower in the PCL‐only groups than in cells cultured on the 3% scaffolds under both non‐IR and IR conditions at all timepoints (Figure 6b, right). *CAT* expression on the 1% scaffold was elevated only at days 7 and 14. Notably, among IR groups, a significant increase in *CAT* expression was observed in the 1% IR scaffold from day 1 to day 14, whereas the 3% IR scaffold showed a transient rise from day 1 to day 7 that did not persist. These results may indicate that moderate adenosine incorporation enhances the antioxidant response of thyroid cells, supporting sustained expression of key enzymatic defences such as *GPX1* and *CAT*, particularly under IR‐induced oxidative stress.

To assess cellular senescence, expression of regucalcin (*RGN*) and cyclin‐dependent kinase inhibitor 2A (*CDKN2A*) which encodes p16, two well‐established markers of stress‐induced growth arrest, was evaluated across all scaffold conditions. *RGN* (Figure 6c, left) expression remained stable over time in all non‐IR groups, indicating the absence of time‐dependent senescence induction under homeostatic conditions. In contrast, all IR groups exhibited a distinct peak in *RGN* expression at day 7, which was significantly higher than in their non‐IR counterparts. By day 14, expression had declined in the PCL‐only and 1% IR scaffolds, whereas it remained elevated in the 0.5% and 3% IR groups. Notably, the 1% IR scaffold showed lower *RGN* expression than the PCL‐only IR group at day 14, while the 3% IR scaffold maintained higher expression levels than its non‐IR equivalent throughout the study. Expression of *CDKN2A* (Figure 6c, right) largely mirrored these findings. The PCL‐only, 0.5%, and 3% IR groups displayed a significant peak in *CDKN2A* expression at day 7 that returned to baseline by day 14, whereas the non‐IR 1% scaffold demonstrated a gradual decrease in expression over time. These results suggest that IR induces a transient senescent response in thyroid cells, which is mitigated most effectively by moderate adenosine incorporation.

Lastly, expression of the apoptosis‐associated protein caspase‐3 (*CASP3*) was evaluated (Figure 6d). Minimal changes were observed in *CASP3* expression among non‐IR cells, except in the 1% scaffold group, which showed a significant decrease from day 1 to day 14. In contrast, the PCL‐only IR group exhibited elevated *CASP3* expression at days 1 and 7 compared to its non‐IR counterpart. Similar increases were observed in the 1% groups at day 1 and in the 0.5% groups at day 14. Notably, non‐IR cells cultured on the 3% scaffold expressed higher *CASP3* levels than those on PCL‐only scaffolds at day 14, potentially indicating cytotoxic effects at this higher adenosine concentration. However, by day 14, the 1% IR scaffold displayed significantly lower *CASP3* expression than both the PCL‐only and 0.5% IR groups. These findings suggest that higher adenosine concentrations may induce cytotoxic stress, while incorporation of 1% adenosine appears to attenuate IR‐associated apoptotic signalling. All IR data is also displayed, normalized to the equivalent control group on the same day containing the same adenosine %, is displayed in Figure S8.

#### 2.3.2 Responses of mTHP‐1 Cells

**FIGURE 7:**
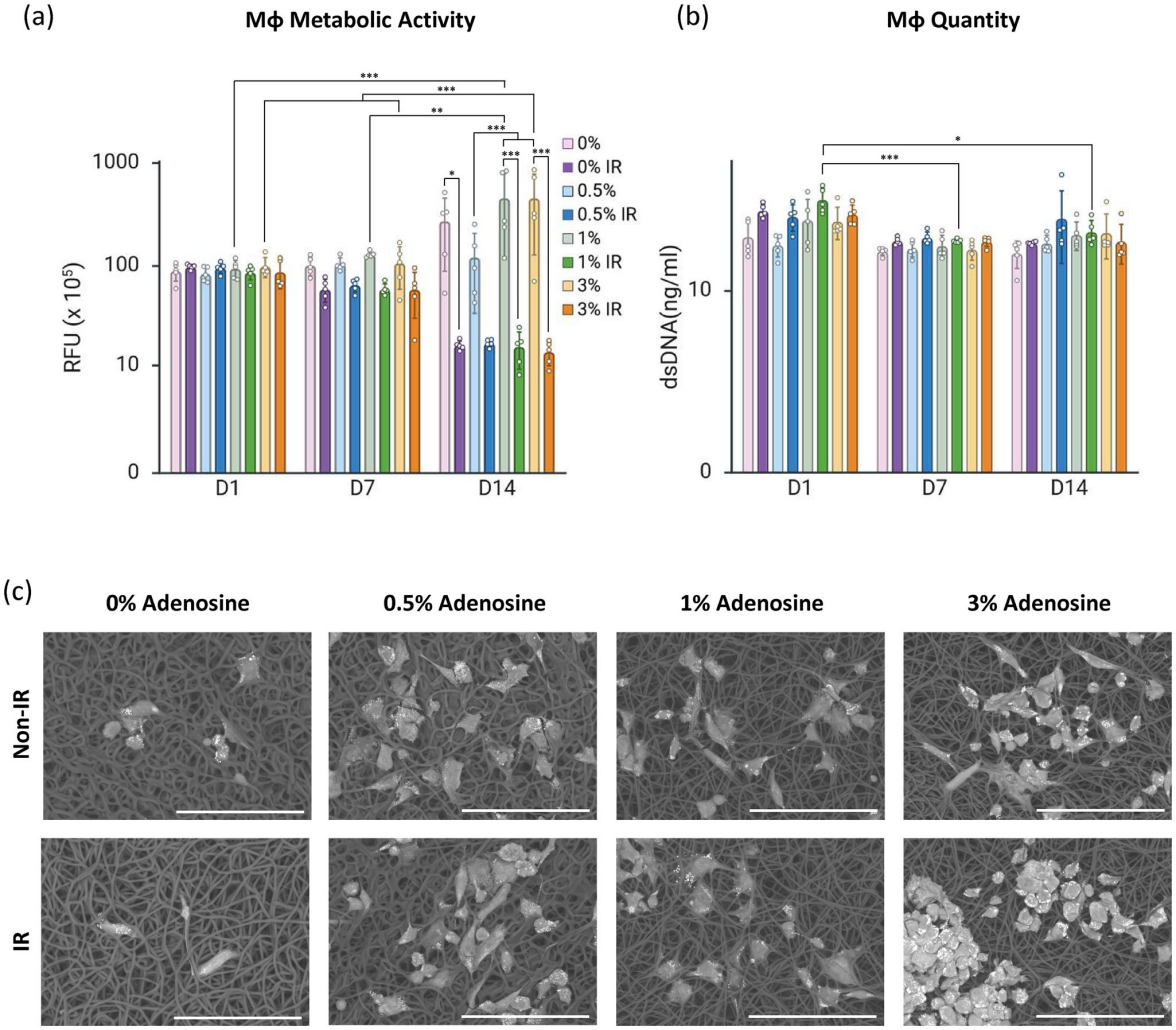
Responses of THP‐1‐derived Mφ to electrospun adenosine scaffolds displaying: (a) metabolic activity measured via the CellTitre blue assay, (b) dsDNA quantification measured via the QuantIT PicoGreen assay and (c) representative osmium‐stained SEM at day 14. N = 5. Statistical analysis: two‐way ANOVA and post hoc Tukey, *p ≤ 0.05, **p ≤ 0.01, ***p ≤ 0.001. Data = mean ± SD. Scale bars = 100 µm.

PCL‐only, 0.5%, 1%, and 3% adenosine scaffolds were seeded with either non‐IR or IR mTHP‐1 cells to assess the polarizing influence of adenosine incorporation on Mφ over a 14‐day period. Only minor variations in metabolic activity were observed across conditions (Figure 7a). Both the 1% and 3% adenosine scaffolds displayed an upregulation in metabolic rate throughout the study, with significantly higher activity than the 0.5% group at day 14. Although no significant changes were detected within the IR groups over time, the metabolic activity of IR cells on the PCL‐only, 1%, and 3% scaffolds was lower than their non‐IR counterparts at day 14. As expected, given that mTHP‐1 cells do not proliferate, DNA content did not increase over the course of the study (Figure 7b). A slight reduction in DNA concentration was noted in the 1% IR group, possibly reflecting limited cell loss or de‐differentiation. Consistent with these findings, SEM imaging revealed uniform cell morphology across all scaffold types, with no visible indicators of apoptosis or necrosis (Figure 7c and Figure S9).

**FIGURE 8:**
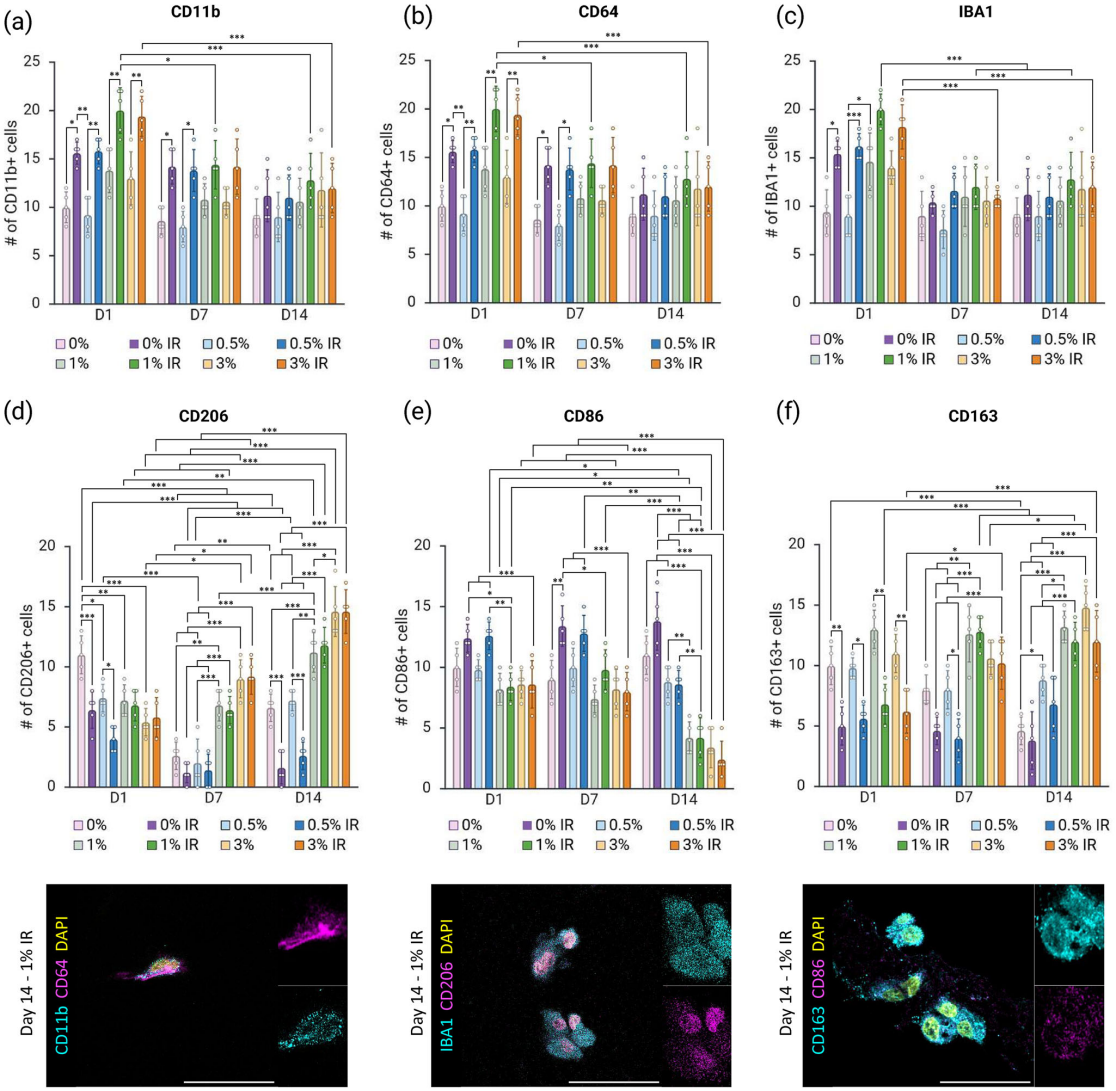
Responses of THP‐1‐derived Mφ to electrospun adenosine scaffolds displaying: IF quantification of cell‐seeded scaffolds stained for (a) CD11b, (b) CD64, (c) ionized calcium‐binding adapter molecule (IBA1), (d) CD206, (e) CD86 and (f) CD163. Representative images of 1% adenosine scaffolds seeded with irradiated cells is shown on the bottom. N = 5. Statistical analysis: two‐way ANOVA and post hoc Tukey, *p ≤ 0.05, **p ≤ 0.01, ***p ≤ 0.001. Data = mean ± SD. Scale bar = 60 µm.

To assess how adenosine‐blended scaffolds influenced Mφ phenotype over time, mTHP‐1‐derived Mφ were evaluated for changes in marker‐positive cell numbers across surface proteins associated with Mφ function and identity. CD11b+ cells remained largely stable across non‐IR scaffolds and timepoints (Figure 8a and Figure S10). In IR conditions, CD11b+ cells were elevated at day 1 across all scaffolds and remained higher at day 7 on PCL‐only and 0.5%, whereas levels on 1% and 3% returned to baseline by day 14. CD64 showed a comparable pattern (Figure 8b and Figure S10). Across non‐IR scaffolds, CD64+ cells were broadly stable over time. Following IR, CD64+ cells were increased at day 1 across scaffolds, with persistence at day 7 on PCL‐only and 0.5% and a return toward baseline by day 14 on 1% and 3%. The overall stability of CD11b and CD64 in non‐IR conditions, together with the transient IR‐associated rise that normalised on 1% and 3%, suggests preserved Mφ identity with attenuation of IR‐driven activation at intermediate‐to‐higher adenosine contents.

IBA1+ cell numbers remained stable across non‐IR scaffold groups throughout the study, with a significant difference observed only between the 0.5% and 1% scaffolds at day 1 (Figure 8c and Figure S11). In contrast, both the PCL‐only and 0.5% IR groups showed elevated IBA1+ cell numbers compared to their non‐IR counterparts at day 1. Over time, IBA1+ cells decreased significantly on the 1% and 3% IR scaffolds, suggesting a gradual reduction in Mφ activation following irradiation. CD206+ cell numbers showed more dynamic changes (Figure 8d and Figure S11). At day 1, the PCL scaffold exhibited the highest number of CD206+ cells, significantly greater than all non‐IR adenosine‐blended conditions. By day 7, CD206+ cells declined sharply on the PCL and 0.5% scaffolds in both non‐IR and IR groups, while increasing on the 3% and 3% IR scaffolds. At day 14, both the 1% and 3% scaffolds supported a higher number of CD206+ cells regardless of IR, with significantly greater counts than the PCL and 0.5% groups. Additionally, the PCL‐only and 0.5% IR groups displayed reduced CD206 expression relative to their non‐IR counterparts at days 1 and 14. Together, these findings indicate that while IBA1 expression remained largely stable, CD206 levels were dynamically modulated over time, suggesting that adenosine concentration may selectively shape Mφ surface marker expression and polarization.

CD86+ cell counts gradually decreased from day 1 to day 14 across all adenosine‐containing scaffolds, regardless of prior cell IR (Figure 8e and Figure S12). In contrast, CD86+ cell numbers remained relatively stable on the PCL‐only scaffold throughout the study, with significantly higher levels than the 1% and 3% scaffolds at day 14. Similarly, the PCL‐only and 0.5% IR groups displayed more CD86+ cells than the 1% and 3% IR groups at day 1, and more than the 3% IR group at day 7. Conversely, CD163+ cell numbers remained relatively constant on the 0.5% and 1% scaffolds over the study period (Figure 8f and Figure S12). While CD163+ cells decreased on the PCL‐only scaffold, they increased on the 3% scaffold from day 1 to day 14. All IR groups exhibited fewer CD163+ cells at day 1 compared to their non‐IR counterparts. This reduction persisted on PCL‐only and 0.5% IR scaffolds, whereas CD163+ cell numbers on the 1% and 3% IR groups recovered to levels comparable with non‐IR conditions. Notably, by days 7 and 14, both the 1% and 3% IR scaffolds supported higher CD163+ cell counts than the PCL‐only and 0.5% IR groups. The opposing trajectories of CD86 and CD163 expression indicate that adenosine incorporation, particularly at intermediate and higher concentrations, promotes a gradual shift in Mφ surface marker profiles, further supporting increased Mφ plasticity and a more reparative phenotype over time.

**FIGURE 9:**
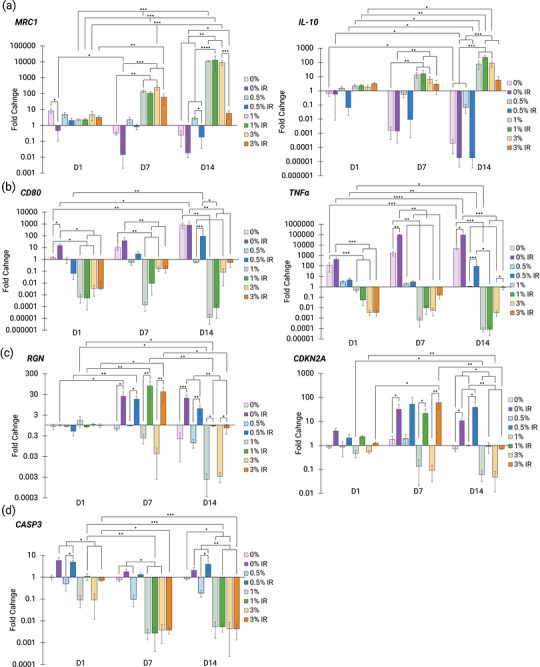
Responses of THP‐1‐derived M φ to electrospun adenosine scaffolds displaying: qRT‐PCR analysis of M φ plasticity‐associated gene expression including (a) *MRC1* (left) and interleukin 10 (*IL‐10*, right), (b) *CD80* (left) and tumour necrosis factor alpha (*TNF*α, right), (c) senescence markers regucalcin (*RGN*, left) and cyclin‐dependent klinase inhibitor 2A (*CDKN2A*) as well as (d) the apoptosis‐associated protein caspase‐3 (*CASP3*) N = 5. Statistical analysis: two‐way ANOVA and post hoc Tukey, *p ≤ 0.05, **p ≤ 0.01, ***p ≤ 0.001. Data = mean ± SD.

To obtain a more comprehensive overview of gene expression, qRT‐PCR was performed for key Mφ markers and cytokines (Figure 9a). Expression of *MRC1*, the gene that encodes CD206, followed a trend consistent with IF quantification, showing progressive upregulation in the 1% and 3% scaffolds throughout the study, and significantly higher expression than the PCL‐only group at days 7 and 14 (left). Similarly, *MRC1* expression increased over time in the 1% IR group, while in the 3% IR group, expression peaked at day 7 before returning to baseline at day 14. Both the 1% IR and 3% IR scaffolds displayed significantly higher *MRC1* expression than the PCL‐only IR group at day 7, and higher than both the PCL‐only IR and 0.5% IR groups at day 14. In contrast, *MRC1* expression was downregulated in the 0.5% group compared to the 1% and 3% scaffolds at day 14. Notably, IR caused a marked reduction in *MRC1* expression in cells cultured on PCL‐only scaffolds at day 1. Expression of *IL‐10* followed a similar but less pronounced pattern (right). *IL‐10* levels increased over time in the 1% and 3% non‐IR scaffolds but declined in the PCL‐only condition. A similar downregulation was observed in the PCL‐only and 0.5% IR groups over the course of the study. At day 14, *IL‐10* expression in the 0.5% group was significantly lower than in the 1% and 3% groups. Conversely, *IL‐10* expression in the 1% IR group increased steadily from day 1 to day 14. Both the 1% and 3% IR scaffolds also showed significantly higher *IL‐10* expression than the PCL‐only IR group at days 7 and 14. These findings suggest that adenosine incorporation, particularly at 1% and 3%, promotes a Mφ phenotype associated with anti‐inflammatory and tissue‐reparative signalling, even following IR.

Contrastingly, assessment of *CD80* expression — a CD28 ligand involved in T cell activation and antigen receptor signalling — revealed a marked increase in both non‐IR and IR PCL‐only groups from day 1 to day 14 (Figure 9b, left). *CD80* expression was significantly higher in the PCL‐only scaffolds than in the 1% and 3% adenosine scaffolds at days 1 and 7, and higher than all other groups by day 14. A similar pattern was observed in the IR groups, where *CD80* expression was consistently lower in the 1% and 3% IR scaffolds compared to the PCL‐only IR scaffold at all timepoints. Interestingly, the 0.5% IR group exhibited significantly higher *CD80* expression than its non‐IR counterpart, as well as the 1% IR group, at day 14. Expression of the pro‐inflammatory cytokine *TNF*α (Figure9b, right) mirrored this pattern, showing a progressive increase in both non‐IR and IR PCL‐only scaffolds over time. The PCL‐only IR group expressed higher *TNF*α levels than the PCL‐only non‐IR group at days 7 and 14. Moreover, *TNF*α expression was significantly higher in the PCL‐only scaffold than in the 1% and 3% scaffolds at day 1 and higher than all groups by days 7 and 14. Similarly, the PCL‐only IR scaffold exhibited greater *TNF*α expression than both the 1% IR and 3% IR scaffolds at all timepoints, as well as higher than the 0.5% IR group at day 7. By day 14, *TNF*α expression in the 0.5% IR group surpassed that of its non‐IR counterpart and the 1% IR scaffold, with a significant upregulation of *TNF*α expression also observed in the 0.5% IR group over the period of study. Additionally, the 3% IR group showed elevated *TNF*α expression compared to the 3% non‐IR scaffold at day 14. These data indicate that adenosine incorporation at 1% and 3% attenuates Mφ pro‐inflammatory activation and cytokine production, suggesting a possible dampening of IR‐induced inflammatory responses.

*RGN* and *CDKN2A* expression were assessed as indicators of persistent cellular stress and irreversible growth arrest in THP‐1‐derived Mφ exposed to IR and varying concentrations of adenosine (Figure 9c). Similar to the thyroid cells, *RGN* expression (left) in THP‐1‐derived Mφ was upregulated in all IR groups at day 7 compared to day 1. Moreover, all IR groups exhibited higher *RGN* expression than their respective non‐IR counterparts at both days 7 and 14. However, both the 1% and 3% IR groups showed a downregulation of *RGN* expression relative to the PCL‐only and 0.5% IR groups at day 14. Interestingly, *RGN* levels decreased over time in both the 1% non‐IR and IR groups. *CDKN2A* expression (right) mirrored this trend in the 1% and 3% non‐IR groups but remained stable in the 1% IR group. In contrast to *RGN*, *CDKN2A* expression was significantly elevated only in IR cells cultured on the PCL‐only, 1%, and 3% scaffolds at day 7, as well as on the PCL‐only and 0.5% scaffolds at day 14. Both the 1% and 3% IR groups displayed a downregulation of *CDKN2A* expression compared to the PCL‐only and 0.5% IR groups at day 14, a pattern also observed in the non‐IR groups. Notably, the 3% IR group showed a transient increase in *CDKN2A* expression at day 7, which returned to baseline by day 14. These findings suggest that adenosine incorporation, particularly at intermediate and higher concentrations, may mitigate IR‐induced senescence and promote a more stress‐resistant Mφ phenotype.

Lastly, expression of *CASP3* was evaluated, with overall expression remaining relatively low across all THP‐1‐derived Mφ (Figure 9d). Differences were only observed between non‐IR and IR groups on the 0.5% scaffold at days 1 and 14, where *CASP3* expression was upregulated following IR. Among non‐IR groups, expression differed only at day 14, with both the 1% and 3% scaffolds displaying reduced *CASP3* levels compared to the PCL‐only group. In contrast, the 1% and 3% IR groups showed a consistent downregulation of *CASP3* expression relative to the PCL‐only and 0.5% IR groups at all timepoints. Moreover, *CASP3* expression steadily decreased over time in IR cells cultured on the 1% and 3% scaffolds. These findings suggest that adenosine incorporation at intermediate and higher concentrations may confer a protective effect against IR‐induced apoptotic signalling in Mφ. All IR data is also displayed normalized to the equivalent control group on the same day containing the same adenosine %, is displayed in Figure S13.

## 3 Discussion

### 3.1 Addressing RIHT: Study Aim and Therapeutic Strategy

Radiation‐induced hypothyroidism (RIHT) is a common yet poorly understood complication of head and neck cancer radiotherapy, with existing treatments limited to hormone replacement. Underlying tissue remodelling, fibrosis and oxidative stress that contribute to long‐term gland dysfunction are not addressed by current therapeutics [4, 75]. Our data reinforce the theory that RIHT goes beyond a simple loss of hormone‐producing cells.

Mφ are key orchestrators of tissue repair, with well‐established roles in modulating ECM turnover, promoting epithelial regeneration, and resolving fibrotic responses following injury [17, 76, 77]. Therefore, modulation of Mφ plasticity presents a promising strategy to support thyroid tissue repair post‐IR injury, mitigating ECM deposition and ROS accumulation. To support this approach, the Mφ compartment of the thyroid was characterized, identifying multiple subpopulations with distinct phenotypes.

Based on these findings, adenosine‐blended electrospun scaffolds were investigated as a biomaterial‐based approach to elicit a combined antioxidant and immunomodulatory effect. It was hypothesized that adenosine‐releasing scaffolds would shift Mφ toward a phenotype conducive for tissue repair, mitigate fibrotic remodelling, and provide a microenvironment able to promote thyroid cell survival and function.

### 3.2 Radiation Injury Induces Long‐Term Thyroid Dysfunction and Structural Remodelling

Using an in vivo murine model of thyroid IR injury, we observed durable structural and functional changes consistent with the prolonged and variable latency of RIHT reported in patients [2, 78]. The reduction in T4, colloid depletion, and follicular rupture with thyrocyte loss confirmed persistent gland dysfunction and support the notion that RIHT arises from sustained tissue‐level injury rather than acute cytotoxicity alone.

While IR‐induced apoptosis is a recognised driver of IR injury, the coinciding upregulation of KRT8 suggests a broader epithelial stress response that contributes to tissue remodelling [79, 80]. Dysregulated KRT8 expression has been implicated in fibrotic processes and EMT, often as a protective response to environmental stressors [81, 82]. The predominance of KRT8 in regions with severe T4 depletion post‐IR is consistent with a chronic stress phenotype and possible fibrotic progression. Together, these observations support a model in which RIHT reflects persistent architectural and cytoskeletal remodelling that impairs tissue homeostasis and aids in explaining the long latency and variable clinical presentation of the disease.

### 3.3 Long‐Term Alterations in Thyroid Myeloid Architecture and Macrophage Plasticity as a Therapeutic Target

To better understand the therapeutic potential of targeting Mφ, the thyroid‐resident compartment was characterized under homeostatic conditions. IF revealed spatially distinct populations expressing IBA1, CD163, CSF1R, and CD11c, indicative of a heterogeneous Mφ pool with features spanning tissue maintenance and antigen presentation. Flow cytometry identified two dominant subsets based on CD206 and CD11c expression: CD206^+^ CD11c^−^ cells enriched for TIM4 and CD163, and CD206^−^ CD11c ^+^ cells lacking these markers. The CD206 ^+^ subset likely plays roles in apoptotic cell clearance and scavenging, consistent with TIM4 and CD163 function in maintaining local tolerance and supporting tissue turnover [68, 83, 84]. Similar subset structures have been described in salivary glands and other mucosal tissues [85]. Functionally, CD206 ^+^ Mφ are often associated with tissue‐supportive, anti‐inflammatory roles, including debris clearance and promotion of wound healing [77, 86, 87]. Although direct functional assays were not performed here, the observed expression profile supports the view that subsets of thyroid Mφ are poised to support repair.

However, in the IR thyroid, a modest but sustained reduction in CSF1R ^+^ cells at later timepoints suggests perturbation of the CSF1R‐dependent Mφ niche. The disruption of the CSF‐1/CSF1R signaling axis can alter the balance between resident and bone‐marrow‐derived myeloid cells [88, 89, 90]. Anti‐CSF1R treatment in non‐IR mice markedly reduced thyroid CD206‐/CD11c+ cell numbers, confirming that this population in the thyroid is heavily dependent on CSF1R signalling, in line with observations in other organs where CSF1R inhibition disrupts tissue and immune homeostasis [62, 91]. IR‐associated loss or reprogramming of this CSF1R‐dependent populations may therefore be an important contributor to chronic inflammation and fibrosis in RIHT.

The broader inflammatory context may reflect canonical injury‐associated cues, including damage‐associated moleculer pattern (DAMP) release and cytokine/chemokine modulation known to follow epithelial disruption and IR [92, 93]. Here, the coexistence of CD11c^+^ and CD163^+^ cells pre‐ and post‐IR may indicate phenotypic diversity within the thyroid myeloid pool, similar to inflamed tissues in other organs, where overlapping Mφ states coexist [94, 95]. Cytokine pathways such as TGF‐β‐driven macrophage–fibroblast interactions are recognized drivers of tissue remodeling and fibrosis across multiple organs and provide a plausible mechanistic framework for the chronic architectural changes observed in the irradiated thyroid [96, 97, 98]. While this provides a general mechanistic framework, future work using single‐cell or lineage‐tracing approaches will be required to delineate recruitment dynamics and macrophage functional states in the IR thyroid.

An increase in CD163 ^+^ Mφ following IR further suggests a shift toward a reparative or anti‐inflammatory myeloid state. CD163, a scavenger receptor enriched on IL‐10– and glucocorticoid‐responsive Mφ, is widely associated with pro‐reparative phenotypes [99, 100]. Although Mφ acutely downregulate CD163 after high‐dose IR in vitro [101], chronic IR injury in lung and other tissues is associated with accumulation of CD163 ^+^ Mφ in fibrotic regions. Their depletion via CSF1R inhibition reduces pathological remodelling [76]. A similar influx occurs in radioiodine‐ablated murine thyroids, where Mφ accumulate in areas of follicular disruption [100, 102, 103]. The increase in CD163 ^+^ Mφ in the IR thyroid may therefore reflect a reparative program driven by persistent epithelial injury, increased apoptosis, and cytokines such as IL‐10, CSF‐1, and TGF‐β, but may also contribute to chronic tissue remodelling and fibrosis alongside KRT8 upregulation.

This data establishes a baseline for thyroid Mφ heterogeneity and provides mechanistic insight into their maintenance and potential roles in tissue homeostasis. Furthermore, IR‐induced remodelling of the myeloid compartment and the presence of reparative subset positions Mφ plasticity as a rational target for supporting thyroid tissue repair. Given their responsiveness to microenvironmental cues, Mφ are well positioned to mediate the effects of an immunomodulatory scaffold. Adenosine‐releasing PCL scaffolds offer a strategy to guide Mφ toward tissue‐supportive states, reduce fibrotic remodelling, and promote regeneration following IR injury.

### 3.4 Composite PCL Scaffolds Are Optimized for Local Delivery of Adenosine and Supportive Tissue Microenvironments

Electrospun scaffolds were designed to deliver adenosine in a controlled manner, enabling localized immunomodulation and antioxidant activity. Previous studies incorporated adenosine into core–shell PCL/PVA or PLA fibres for bone regeneration and analgesia [57, 58]. Here, adenosine was blended directly with PCL, achieving up to 3% (w/v) loading while maintaining continuous, bead‐free fibres at concentrations ≤ 3%. This represents relatively high drug loading compared with typical electrospun systems. Structural studies of A2A and A2B receptors show that adenosine's purine ring and ribose sugar form key hydrophobic and hydrophilic contacts within the receptor binding pocket [104, 105, 106, 107]. Blend electrospinning therefore offers the advantage of preserving critical binding motifs in the active compound, whereas covalent immobilisation could occlude receptor‐binding surfaces and blunt downstream signalling.

PCL and adenosine showed high solubility in HFIP, a solvent well suited to electrospinning [108]. Morphology remained favourable up to 3% adenosine, whereas 4% yielded flattened ribbons consistent with solvent‐driven phase separation and jet collapse [109]. Subtle changes in fibre surface topography and diameter distribution at 0.5% adenosine suggest non‐linear effects of concentration on jet stability, potentially through liquid–liquid phase separation.

The composite scaffolds exhibited fibre diameters within the range previously identified as optimal for Nthy‐ori 3‐1 proliferation and adhesion (1.1–2.9 µm) [26]. Interestingly, higher adenosine loading increased scaffold porosity despite a reduction in mean fibre diameter, which is advantageous for cellular infiltration and nutrient/waste exchange [74]. Porosity has also been associated with Mφ polarization. More open architectures encourage immunomodulatory profiles and secretion of pro‐regenerative factors [22, 110].

Mechanically, increased adenosine content decreased stiffness and strength, in contrast to prior core–shell designs, where adenosine increased modulus [57]. Given that PCL is substantially stiffer than native thyroid tissue, this reduction may bring the scaffold closer to physiological compliance, as the normally soft and elastic thyroid may become stiffened following IR as a result of increases in fibrotic markers [35, 111, 112].

These characteristics, coupled with adenosine release profiles that provide an early burst followed by sustained delivery, suggest that composite PCL/adenosine scaffolds are well suited to address the acute ROS surge and prolonged immune dysregulation arising during RIHT. The following describes how these properties translate into functional support for thyrocytes and Mφ.

### 3.5 Adenosine Scaffolds Support Thyrocyte Viability and Function in a Dose‐Dependent Manner

The effects of adenosine‐blended scaffolds on thyrocyte viability, epithelial architecture, and function were examined over 14 days under IR and non‐IR conditions. While electrospun PCL alone supports epithelial/endocrine attachment through favourable porosity and mechanics [26, 35], adenosine adds receptor‐mediated bioactivity [113, 114]. Our data show that adenosine's impact is strongly dose‐dependent.

On PCL‐only scaffolds, Nthy‐ori 3‐1 cells maintained endocrine identity and modest junctional maturation but showed transient αSMA induction, consistent with mild myofibroblast‐like activation [115, 116, 117, 118, 119]. IR reduced proliferation and expression of *TPO* and *NKX2‐1*, reflecting known radiosensitivity [64]. At 0.5% adenosine, functional gains were limited. *TPO* and *NKX2‐1* remained lower than in PCL or 1% scaffolds, suggesting insufficient receptor engagement [120, 121].

In contrast, 1% adenosine provided a clear functional advantage. This scaffold supported sustained proliferation, increased Tg and Ki67 expression, and enhanced epithelial cohesion as indicated by higher ZO‐1, ECAD and KRT8 [122, 123]. αSMA remained low, consistent with adenosine's anti‐fibrotic activity [124]. Importantly, *TPO* and *NKX2‐1* expression were preserved in IR cells on 1% scaffolds, suggesting partial protection of function [125, 126]. These effects are compatible with A2A/A2B signalling, known to stabilise mitochondrial function and support epithelial resilience [127, 128]. At 3%, adenosine initially improved epithelial markers but benefits were not sustained; late declines in *TPO* and *NKX2‐1* suggest receptor desensitisation or pathway imbalance under chronic high‐dose stimulation [129, 130].

Overall, these results identify a therapeutic window in which 1% adenosine optimally balances thyrocyte viability, epithelial architecture and functional gene expression while mitigating IR‐induced dysfunction. Compared with other bioactive scaffolds designed for epithelial or endocrine repair, the performance of the 1% scaffold is comparable to IL‐10/IL‐4‐functionalised matrices or EGF‐based coatings, which enhance organisation and repair but often require exogenous protein supplementation [47, 131, 132, 133]. Adenosine offers an endogenous, receptor‐driven signal that can act on multiple cell types without continuous factor delivery. Antioxidant polyphenol‐loaded scaffolds, such as quercetin‐based systems provide important precedent for protecting epithelia from IR [49, 134], but rely largely on chemical scavenging rather than adaptive receptor‐mediated programmes. Thus, adenosine‐blended scaffolds represent a biologically integrated approach that couples structural support with intrinsic cytoprotection.

### 3.6 Adenosine Enhances Antioxidant Enzyme Activity, Mitigating Oxidative Stress

Oxidative stress emerged as a key feature of IR injury in the thyroid, with persistent nuclear ROS signals and KRT8 upregulation supporting a model of chronic oxidative and cytoskeletal stress [81, 135, 136]. Although ROS typically have very short half‐lives [137, 138], their prolonged detection post‐IR suggests ongoing production, likely involving immune cells such as pro‐inflammatory Mφ that generate high levels of ROS and can perpetuate damage [139, 140, 141]. These observations align with broader evidence that oxidative stress and fibrosis are central to RIHT pathogenesis [142, 143].

Adenosine scaffolds increased the expression of key antioxidant enzymes. In thyrocytes, *GPX1* was elevated on 1% and 3% scaffolds under both IR and non‐IR conditions, while *CAT* increased particularly at later timepoints and under IR in the 1% group [144, 145]. Given their complementary roles in detoxifying peroxides and maintaining redox balance, these changes indicate that adenosine enhances intrinsic antioxidant defences. Compared with other strategies, such as quercetin‐ or N‐acetylcysteine‐based biomaterials, which provide direct radical scavenging or transient boosting of intracellular defences [134, 146, 147, 148], adenosine engages A2A/A2B receptor pathways that simultaneously dampen inflammation and upregulate cytoprotective programmes [127, 130]. The sustained *GPX1*/*CAT* response observed here is consistent with such receptor‐mediated, adaptive regulation and supports adenosine as a rational antioxidant component for RIHT‐targeted scaffolds.

### 3.7 Macrophage Activity is Regulated by Adenosine‐Blended Scaffolds

Adenosine‐blended scaffolds also modulated Mφ plasticity. THP‐1‐derived Mφ maintained lineage markers (CD11b, CD64, IBA1) across conditions, indicating that adenosine did not destabilize cell identity. However, scaffold composition and IR status jointly influenced activation profiles. On PCL‐only scaffolds, IR Mφ showed increased CD86, reduced CD206/CD163, and elevated *CD80* and *TNF*α, indicative of a sustained pro‐inflammatory state [76, 117, 149, 150, 151]. These patterns are compatible with chronic inflammatory activation in a biomaterial context lacking immunomodulatory cues.

Low‐dose adenosine (0.5%) only partially attenuated these responses. Post‐IR *MRC1* and *IL‐10* remained low while *CD80* and *TNF*α stayed elevated. This suggests sub‐threshold receptor engagement. At 1%, adenosine drove a coordinated shift toward a regulatory phenotype, with increased CD206 and CD163, decreased CD86, upregulated *MRC1* and *IL‐10*, and suppression of *CD80* and *TNF*α, even after IR. These findings align with well‐established roles of A2A/A2B activation in suppressing pro‐inflammatory NF‐κB signalling and promoting IL‐10–rich repair programmes [53, 152, 153]. At 3%, similar trends were observed, likely reflecting receptor saturation rather than additional benefit; very high stimulation may risk desensitization [152].

The magnitude and direction of Mφ modulation are comparable to cytokine‐functionalised or metabolite‐loaded scaffolds that promote injury resolution. IL‐10‐ or IL‐4‐modified fibres, and polyphenol‐ or itaconic‐acid‐functionalised membranes, have been shown to enhance reparative Mφ polarization and reduce inflammatory signalling [45, 47, 51, 131, 154]. Local adenosine delivery via injectable microgels has similarly been reported to support tissue regeneration and Mφ reprogramming [155]. Our data indicate that adenosine‐blended PCL scaffolds achieve comparable immunomodulatory performance through a physiologically relevant, receptor‐driven mechanism, positioning them as a credible platform for restoring immune homeostasis and promoting repair in the irradiated thyroid [97, 155].

### 3.8 Adenosine Modulates Cellular Senescence and Apoptotic Pathways Following Irradiation

Ionizing radiation is a potent inducer of stress responses that culminate in senescence and apoptosis. *RGN* and *CDKN2A* are indicators of stress‐induced growth arrest, whereas *CASP3* is a central executioner of apoptosis [156, 157, 158]. IR in this study induced dynamic changes in these markers in both thyrocytes and Mφ, and scaffold composition shaped the persistence or resolution of these responses.

In Nthy‐ori 3‐1 cells, *RGN* and *CDKN2A* peaked at day 7 after IR but declined by day 14 on 1% adenosine scaffolds, whereas elevations persisted with lower or higher adenosine contents. *CASP3* was initially higher on PCL‐only IR scaffolds and fell on 1% IR scaffolds over time. THP‐1 Mφ showed a similar pattern, with IR‐associated increases in *RGN* and *CDKN2A* at day 7 that were attenuated by day 14 on 1% and 3% scaffolds, alongside overall lower *CASP3* than on PCL‐only or 0.5% scaffolds. These findings indicate that within a concentration window, adenosine reduces sustained senescence‐like signalling and apoptotic propensity in both epithelial and immune cells.

Mechanistically, these observations are compatible with A2A/A2B signalling restraining upstream drivers of senescence and apoptosis via suppression of inflammatory and oxidative stress pathways [121, 127]. The scaffold‐dependent normalisation of *RGN*/*CDKN2A* and down‐modulation of *CASP3* on 1% adenosine supports a receptor‐mediated cytoprotective effect. This aligns with emerging biomaterial strategies that incorporate IFI6, miRNAs, or stem‐cell‐derived factors to attenuate IR‐induced apoptosis and improve tissue repair in skin and other tissues [159, 160, 161, 162, 163, 164]. In this context, adenosine‐blended scaffolds extend such concepts to a thyroid‐mimetic system, suggesting that receptor‐engaging biomaterials can form part of a broader toolkit for limiting IR‐associated senescence and cell loss.

### 3.9 Limitations and Future Directions

This study provides insights into thyroid responses to IR and demonstrates the potential of adenosine‐blended electrospun scaffolds, but several limitations must be acknowledged. Scaffold composition and release kinetics also require further optimization and characterisation, including nanoscale adenosine distribution and extended release profiling. While morphology, porosity, mechanics, hydrophilicity, FTIR and release were comprehensively assessed here [26, 165, 166, 167, 168], additional techniques such as TEM, Raman spectroscopy, XPS, TGA, and DSC would refine understanding of polymer–drug interactions and guide further tuning. As this study relied on in vitro testing with IR‐cells, long‐term in vivo evaluation of scaffold integration, immune modulation, and endocrine restoration should be conducted once the above listed parameters have been fully investigated and optimized.

PCL's intrinsic hydrophobicity, exacerbated by adenosine blending, may limit adhesion compared to natural polymers [169], although this did not impede the in vitro responses observed. Surface treatments such as plasma modification could be explored to further tailor scaffold–cell interactions if needed [170]. Extending adenosine release analyses beyond 105 h and using more sensitive quantification methods (e.g., HPLC) would help align release profiles with observed cellular responses.

Discrepancies between PCR and IF for some markers, including CD206 and its transcript *MRC1*, highlight the importance of combining mRNA and protein‐level analyses and considering cell number effects [171, 172]. Future work should incorporate more functional assays of Mφ and thyrocyte behaviour, including phagocytosis, cytokine secretion, and hormone output.

Finally, CSF1R signalling emerges as an additional tunable axis. The CD206 ^−^ thyroid‐resident Mφ subset was CSF1R‐dependent, and prior studies have successfully harnessed CSF1R modulation to deplete or reprogramme Mφ [173, 174, 175, 176]. Combined strategies that integrate CSF1R‐targeted approaches with adenosine‐functionalised scaffolds may offer synergistic control over Mφ maintenance, recruitment, and function in the irradiated thyroid.

In summary, while further optimisation and in vivo validation are required, this work lays a strong foundation for advancing adenosine‐blended electrospun scaffolds as a targeted, immune‐guided strategy for restoring thyroid structure and function following IR, directly addressing mechanisms of oxidative stress, Mφ dysregulation, and maladaptive tissue remodelling that underlie RIHT.

## 4 Conclusion

Radiation‐induced hypothyroidism (RIHT) continues to represent a major clinical limitation of radiotherapy, with no existing therapies capable of preventing or reversing the oxidative stress, chronic inflammation, and fibrosis that drive thyroid dysfunction. This work demonstrates that electrospun polycaprolactone (PCL) scaffolds functionalized with adenosine provide a dual‐action strategy for modulating post‐irradiation tissue responses through both immunoregulatory and cytoprotective mechanisms.

The data show that adenosine incorporation into PCL scaffolds promotes Mφ polarization toward a CD206+/CD163+ phenotype while suppressing pro‐inflammatory markers such as CD86, *CD80*, and *TNF*α, thus establishing a more regenerative microenvironment. Concurrently, thyrocyte viability, epithelial organization, and thyroid‐specific function are preserved, accompanied by upregulation of key antioxidant enzymes *GPX1* and *CAT* and downregulation of senescence‐ and apoptosis‐associated genes, including *RGN*, *CDKN2A*, and *CASP3*. Among all formulations, the 1%(w/v) adenosine scaffold provides the optimal balance of bioactivity, structural integrity, and cellular compatibility, supporting a microenvironment that counteracts radiation‐induced oxidative injury and fibrotic remodelling.

These findings establish adenosine‐blended scaffolds as a promising therapeutic platform for restoring thyroid homeostasis after radiotherapy. Future studies should integrate advanced material characterizations and in vivo models to refine adenosine release kinetics and assess endocrine recovery under physiological conditions. With further optimization, this approach could offer a single‐intervention, biomaterial‐based therapy capable of re‐establishing thyroid function through targeted immune modulation and tissue regeneration, reducing the lifelong dependence on hormone replacement therapies.

## 5 Experimental Section

### 5.1 Mouse Studies

All procedures adhered to the Animal Research. The in vivo Experiments (ARRIVE) guidelines and were approved by the U.K. Home Office, performed by the project license holder PB5FC9BD2 (Emmerson). Male and female C57BL / 6J mice, 8–10 weeks old, were used. All mice were randomized into experimental groups and the researcher conducting the imaging and subsequent analysis was blinded to the experimental conditions.

### 5.2 Model of Thyroid IR Injury

Before γ‐irradiation, mice were anaesthetized with 80 mg/kg of ketamine (Ketavet) and xylazine in sterile water (Thermo Fisher Scientific) by injection IP. The IR of the mice was carried out using a single ^137^Cs source (10 Gy) in a Shepherd Mark‐I‐68A irradiator (JL Shepherd & Associates) with only the neck exposed and the head and body shielded by lead (Figure 1a), as previously described [177]. After 20 min of anaesthesia, mice were administered 1 mg/kg atipamezole (Antisedan; reversal agent) and allowed to recover in a heated cabinet before returning to normal housing. Subsequently, mice received a soft diet and DietGel (Clear H_2_O) ad libitum to mitigate the effects of gland injury. Mice utilized for DCFDA staining were sacrificed 28‐days post‐IR via sodium phentobarbital by IP and confirmed by cervical dislocation. All other animals were sacrificed using CO_2_ overdose. Induction of RIHT was confirmed by the lack of T4 via immunofluorescent analysis. Non‐IR control mice were not anaesthetized as a refinement, since published research has demonstrated no difference between control anaesthetized and control non‐anaesthetized animals [62, 177].

### 5.3 Anti‐CSF1R Treatment

Anti‐CSF1R antibodies (AFS98) were purified from culture supernatant of AFS98 hybridoma as previously described [62, 178]. Anti‐CSF1R antibodies (2 mg/ml) were administered through an intravenous injection over three consecutive days at 250 µg per mouse per day and assessed the day after the last administration.

### 5.4 Histology

Thyroid glands were resected and fixed for 4–6 h in 4% paraformaldehyde (PFA, Thermo Fisher Scientific) at room temperature (RT) with constant agitation. Tissue utilized for Mφ staining was fixed for 1 hr in antigen fix. This was followed by PBS washes (3 × 10 min, Merck) and incubation in 30% sucrose before embedding in optimal cutting temperature compound (OCT, Leica) over dry ice. 10 µm sections were cut using a cryostat (Leica) and stored at –80 °C for Mφ staining and at –20 °C for all other tissue staining.

The OCT‐embedded sections were rehydrated in decreasing concentrations of ethanol in water prior to incubation in haematoxylin nuclear stain for 2.5 min, 1% acid alcohol, and Scott's tap water. Sections were counter‐stained with eosin for 5 s and dehydrated in increasing concentrations of ethanol in water. Sections were mounted with ProLong Gold antifade mountant (Invitrogen) and examined under a bright‐field microscope.

### 5.5 Single‐Cell Suspension

Excised thyroid glands underwent mechanical digestions in a GentleMACS Dissociator (Miltenyi Biotec) in 500 µl RPMI 1640 (+2% Fetal Calf Serum). 50 µl collagenase II (23 mg/ml) (Sigma–Aldrich), 50 µl hylaruonidase (40 mg/ml) (Sigma–Aldrich) and 250 µ l of 50 mM CaCl_2_ were added and incubated at 60 °C for 60 min. This was followed by manual filtration (70 µm) (ThermoFisher) and centrifugation (250 g). Cells were resuspended in 0.5 ml of Dulbecco's Modified Eagle's Medium (10% Foetal Bovine Serum, 2 mM L‐Glutamine, 1% Antibiotic‐Antimycotic) (Sigma–Aldrich) per gland.

### 5.6 DCFDA Staining

Live cells isolated from murine thyroids (N = 6) were centrifuged, resuspended in 1 ml PBS and stained with the DCFDA assay (Abcam) according to the manufacturers protocol. Briefly, cells were incubated with DCFDA for 30 min, which was a chemical oxidized by cellular reactive oxygen species. The staining solution was removed via centrifugation and cells were washed with the kits' buffer prior to imaging on slides using a Nikon TiE wide‐field microscope.

### 5.7 Flow Cytometry

Flow cytometry followed a previously described protocol [62]. Briefly, equal numbers of cells were stained with 1:1000 anti‐CD16/32 (2.4G2, BioLegend) in FACS Buffer (100 µl of HBSS, 1% BSA, 2 mM EDTA) for 15 min to reduce non‐specific immunoglobulin G receptors binding. Cells were stained with conjugated antibodies, listed in Table 1, for 30 min at 4 °C in the dark. Samples were washed with FACS buffer and centrifuged at 400 g (5 min at 4 °C) before resuspension in 300 µl of FACS buffer. Single‐stain controls were prepared using OneComp Beads (Thermo Fisher Scientific). Fluorescence minus one (FMO) controls were prepared using cells only. Counting beads (Thermo Fisher Scientific) were included to calculate absolute numbers. 4′,6‐Diamidino‐2‐phenylindole (DAPI; Sigma–Aldrich) and Zombie Fixable Viability Dye (BioLegend) was used as a dead cell marker. Samples were analyzed using an LSRII (BD Biosciences). Data n ≥ 7 was obtained from two independent experiments and analyzed using FlowJo v.10.10.0.

**TABLE 1:** Anti‐mouse flow cytometry antibodies.

Antibody	Flurophore	CAT #	Vendor
CD45	BV510	103138	Biolegend
Live/dead	DAPI		
CD11b	APC Fire 750	101262	Biolegend
CD11c	BV785	117336	Biolegend
Ly6C	PerCP Cyanine5.5	128012	Biolegend
Ly6G	FITC	127606	Biolegend
SiglecF	FITC	130‐112‐178	Miltenyi
MHC‐II	AlexaFluor 700	107622	Biolegend
F4/80	APC	17‐4801‐82	Invitrogen
CD206	eFluor450	48‐2061‐80	Invitrogen
CD163	PE	156704	Biolegend
CX3CR1	Biotin	149005	Biolegend
Tim4	PE Cy7	130010	Biolegend

### 5.8 Electrospinning

Solutions consisting of 10% (w/v) PCL (average Mn = 80 000 g/mol) and 0, 0.5%, 1%, 3% or 4% (w/v) adenosine (both Sigma–Aldrich) in hexafluoroisopropanol (HFIP) (Manchester Organics) were prepared in a glass vial, and placed on a tube roller overnight at room temperature.

Scaffolds were fabricated using an EC‐DIG electrospinner (IME Technologies; software version 1.21) under ambient temperature and humidity, as previously described [168, 179]. The solutions were loaded into 20 mL syringes and placed into a syringe pump (Harvard Apparatus). Initial electrospinning parameters were chosen based on methods from [26], then optimised for consistent morphologies and similar fibre diameters. A total of 6 ml of solution was spun at a feed rate of 1 ml/h using a needle with a 0.3 mm internal diameter. Solid fibres were collected on cylindrical mandrel covered with aluminium foil, set to a rotational speed of 250 RPM. Additional electrospinning parameters are outlined in Table 2. Scaffolds were left to dry in a fume hood for 48 h prior to testing. Scaffolds were referred to throughout via the adenosine % (w/v) concentration within the polymer solution.

**TABLE 2:** Electrospinning parameters.

Adenosine % (w/v)	Positive voltage (kV)	Negative voltage (kV)	Distance between needle tip and mandrel (cm)
0	14	4	14
0.5	13	4	12
1	13	4	12
3	13	4	12
4	12	4	12

### 5.9 Scaffold Characterization

#### 5.9.1 Scanning Electron Microscopy

10 mm diameter circular scaffolds were punched using a biopsy punch, lifted from the aluminium foil using 70% ethanol, and left to dry in a fume hood overnight. Samples were sputter coated with gold using an Emscope SC 500 Sputter Coater (Emscope) then imaged with a TM4000 tabletop scanning electron microscope (SEM) (Hitachi) using a 10 mm working distance, 10 kV accelerating voltage, and back scattered electron detection mode.

Average fibre diameters were obtained using ImageJ. The image scale bar was utilised to determine the number of pixels per unit length, and straight lines were superimposed across fibre diameters (N = 50).

#### 5.9.2 Fourier‐Transform Infrared Spectroscopy

10 mm diameter circular scaffolds were punched, lifted from aluminium foil, and left to dry in a fume hood overnight. Spectra between 4000 and 400 cm^−1^ were taken using a Nicolet iS 10 Spectrometer equipped with OMNIC Spectra software (Thermo Fisher Scientific).

#### 5.9.3 Mechanical Testing

Mechanical properties in tension were obtained using an Instron 3367 tensile testing machine (Instron) equipped with a 50 N load cell. Rectangular samples of 10 mm × 40 mm were prepared and lifted from foil using 70% ethanol, prior to being left to dry in a fume hood overnight. Scaffold thickness was measured using an electric multimeter with an accuracy of ± 0.001 mm, then samples were stretched until failure at a rate of 50% strain/minute, using a starting gauge of 20 mm and pre‐load of 0.01 N (N = 4).

Young's Modulus, *E*, was calculated according to Equation (1), where σ denotes stress, ε denotes strain, *F* is force, *A* is scaffold cross‐sectional area, *L_0_* is the initial length and Δ *L* is change in length.

(1) $$E=\frac{\sigma }{\varepsilon }=\frac{FL_{0}}{A\Delta L}$$

The elongation at break is defined as the change in length, Δ *L*, divided by the original length, *L_0_*, presented in Equation (2).

(2) $$\text{Elongation at break}\left(\%\right)=\frac{\Delta L}{L_{0}}$$

#### 5.9.4 Porosity & Swelling

10 mm diameter circular scaffolds were punched, lifted from aluminium foil, then left to dry in a fume hood for 24 h. Scaffold thickness was measured using an electric multimeter with an accuracy of ± 0.001 mm, and dry weights, *W_d_*, taken to an accuracy of ± 0.01 mg using an XS205 analytical balance (Mettler Toledo).

The porosity of each sample (N = 4) was calculated via Equation (3), whereby theoretical densities, were determined using the mass fractions of PCL and adenosine, taken as 1.145 and 1.387 g/cm^3^ at 25 °C [180].

(3) $$\text{Porosity}=1-\frac{\rho_{measured}}{\rho_{theoretical}}\;\text{x 100}$$

Where ρ equals the density. To investigate scaffold swelling characteristics, samples (N = 4) were immersed in 1 ml of PBS (Merck) and incubated for 48 h at room temperature. Scaffolds were removed from the PBS, blotted once on each side with lint‐free tissue to remove excess water, then re‐weighed to obtain wet weights *W_w_*. Water uptake was calculated according to Equation (4), expressed as the percentage increase in weight.

(4) $$\text{Water Uptake}\left(\%\right)=\frac{W_{w}-W_{d}}{W_{d}}\;\text{x 100}$$

#### 5.9.5 Water Contact Angle

Scaffold hydrophilicity was determined using the sessile drop method (N = 4). A 5 µl droplet of de‐ionised water was placed onto dry scaffolds and recorded using a DMK 41 AU02 monochrome camera and Bandicam 2025 software. Analysis was undertaken using the Water Contact Angle plug‐in within ImageJ.

#### 5.9.6 Release Kinetics

The release profile of adenosine from the electrospun scaffolds was determined using UV–vis spectroscopy. Briefly, 10 mm diameter circular scaffolds (N = 4) were punched, lifted from aluminium foil, then left to dry in a fume hood for 24h. Scaffolds were immersed in 1 ml of PBS and placed into a static incubator set to 37 °C. At pre‐determined time intervals, 110 µl of solution was removed and stored at –80 °C, and replaced with an equal volume of fresh PBS.

100 µl of solution was transferred to a clear bottomed well plate and absorbance at 276 nm was measured using a Clariostar Plus Microplate Reader (BMG Labtech). To determine the concentration of adenosine at each timepoint, a concentration curve of adenosine in PBS between 0 and 360 ng/mL was prepared.

### 5.10 Cell Culture

The human immortalized thyrocyte cell line Nthy‐ori 3‐1 cells, obtained at passage 0 from the European Collection of Authenticated Cell Cultures (ECACC), were expanded to passage 4 before use. Cells were cultured with RPMI‐1640 medium (10% Foetal Bovine Serum, 2 mM L‐Glutamine, 1% Anti‐Anti) (Sigma–Aldrich) as per manufacturer's directions (5% CO_2_, 37 °C).

THP‐1 cells were expanded to passage 3 in RPMI‐1640 (20% Foetal Bovine Serum, 2mM L‐Glutamine, 1% Anti‐Anti) (Sigma–Aldrich). FBS concentration was decreased to 10% for expansion to passage 6. Cells were exposed to 5 ng/mL phorbol 12‐myristate 13‐acetate (PMA) for 24 h. Non‐adherent cells were removed and fresh complete media was applied to allow for a 72 h rest period.

### 5.11 In Vitro IR

Once grown to confluency, mTHP‐1 and Nthy‐ori 3‐1 cells were irradiated using a single ^137^Cs source (10 Gy) in a Shepherd Mark‐I‐68A irradiator (JL Shepherd & Associates). Media volume was doubled prior to transport to negate cell lifting and nutrient deficiency. Post‐IR, fresh complete media was added and cells were allowed a rest period of 2 h prior to scaffold seeding.

### 5.12 Scaffold Seeding and In Vitro Assessment

#### 5.12.1 Scaffold Seeding

8 mm scaffold biopsy punches (Peddinghaus) were sterilized in 70% ethanol and incubated with 1% Antibiotic–Antimycotic in PBS (5% CO_2_, 37 °C) for 12 h followed by 1 h in complete media. Scaffolds were seeded with Nthy‐ori 3‐1 cells and THP‐1‐derived Mφ to assess cell:material interactions and overall biocompatibility. These two cell types were chosen to provide complementary perspectives on thyroid epithelial and immune compatibility. Biocompatibility assessment included evaluation of cell morphology, viability, and phenotype following culture on both PCL and adenosine‐blended scaffolds. This methodological design is consistent with established biomaterials testing frameworks [181, 182]. Cells were lifted at 80% confluency and drip seeded at a density of 10 000 cells/scaffold and 100 000 cells/scaffold, for Nthy‐ori 3‐1 cells and mTHP‐1 cells, respectively, as previously described [183]. In brief, 20 µl of cell suspension was seeded onto the scaffolds; following 30 min incubation (5% CO_2_, 37 °C), 30 µl of complete medium was added, and topped up to 500 µl after an additional 1h incubation. Media was changed after 24 h and every 48 h subsequently. Seeded scaffolds were assessed at 24 h, 7 days and 14 days.

For comparative analyses, experiments were performed using both IR and non‐IR cells cultures. IR was conducted outside the incubator, and non‐IR controls were removed for an equivalent period to minimise handling‐related variability. A parallel experiment in which non‐IR cells remained under standard culture conditions, thereby avoiding additional environmental stressors, was conducted using the same methodology and biocompatibility assessment as listed below, and is showcased in the Figures S14– S19.

#### 5.12.2 Metabolic Assay

The CellTitreBlue (CTB, Promega) assay was performed as previously described [184]. Briefly, viable cells were convert the dye resazurin to fluorescent resorufin, whilst non‐viable cells cannot complete this process due to a loss of metabolic activity. Scaffolds were transferred to a new plate and 500 µl of media‐CTB (ratio 4:1) was added, followed by 3 h incubation (37 °C, 5% CO_2_) protected from light. Fluorescent intensity of 100 µl solution was measured in a black well plate with a Modulus II microplate reader (N = 5) at an excitation wavelength of 525 nm and emission wavelength of 580–640 nm. All data has been represented with removed background fluorescence.

#### 5.12.3 Cell Proliferation

Scaffolds were freeze‐dried for 12 h before a 48 h enzymatic digestion (2.5 units papain/mL, 5 mM Cysteine, 5 mM ethylenediaminetetraacetic (EDTA) in DNase‐free water) at 65 °C with agitation. The Quant‐IT PicoGreen dsDNA assay (Life Technologies) was performed to quantify the DNA content, as according to manufacturer's instructions. Fluorescent intensity measurements were read in a Modulus II microplate reader (N = 5) at an excitation wavelength of 480 nm and emission wavelength of 510–570 nm. A standard lambda dsDNA curve of graded known concentrations was used to calibrate fluorescence intensity vs. dsDNA concentration.

#### 5.12.4 Osmium Staining

Scaffolds were washed with PBS (3 × 10 min) and fixed with 4% Glutaraldehyde for 12 h before storage (4 °C) in PBS. 1% osmium in deionized water was applied to the scaffolds for 30 min, followed by 4 × 5 min washes with deionized water. Samples were dehydrated with sequential ethanol washes (30–100%) and hexamethyldisilazane (HMDS) before storage with light protection. Cell and scaffold interactions were studied with a Hitachi TM4000 SEM and the software ImageJ.

#### 5.12.5 Quantitative Polymerase Chain Reaction (q‐PCR)

RNA from samples was extracted using the phenol–chloroform method [185, 186]. Briefly, cell‐seeded scaffolds (N = 5) were lysed with Tri‐Reagent (Invitrogen) and stored at –80 °C until further use. Chloroform was added, and samples centrifuged (120 000 g, 10 min) following vigorous vortexing. The aqueous RNA supernatant was collected and purified using the RNeasy spin column system (Qiagen) as per manufacturer's instruction, and eluted with 20 µl nuclease‐free water (Invitrogen). Nucleic acid concentration was determined using a Nanodrop Spectrophotometer (Thermo) and equalised by addition of nuclease‐free water. Complementary DNA (cDNA) was produced using primers displayed in Table 3 and the ImProm‐II Reverse Transcription kit (Promega) as according to manufacturer's instructions, and stored at –20 °C until use. Quantitative real‐time PCR was conducted using the Go‐Tag qPCR system (Promega) and a ProFlex PCR system (Applied Biosystems). Glyceraldehyde 3‐phosphate dehydrogenyase (GAPDH) was chosen as a housekeeping gene and results were normalized to cells grown on tissue culture plastic. Fold change was calculated using the 2^−ΔΔCt^ method.

**TABLE 3:** Primer sequences used for qRT‐PCR.

**Gene**	**Sequence**	
*TPO*	Forward	5'TTGTACAACGGGTTCCCACT
	Reverse	5'GGAGGTCAGAATAGCGGTCA
*NKX2‐1*	Forward	5'AGCACACGACTCCGTTCTC
	Reverse	5'GCCCACTTTCTTGTAGCTTTCC
*GPX1*	Forward	5'CAGTCGGTGTATGCCTTCTCG
	Reverse	5'GAGGGACGCCACATTCTCG
*CAT*	Forward	5'TGTTGCTGGAGAATCGGGTTC
	Reverse	5'TCCCAGTTACCATCTTCTGTGTGTA
*MRC1*	Forward	5'CACCATCGAGGAATTGGACT
	Reverse	5'ACAATTCGTCATTTGGCTCA
*IL‐10*	Forward	5'GATGCCTTCAGCAGAGTGAA
	Reverse	5'GCAACCCAGGTAACCCTTAAA
*CD80*	Forward	5'GAAGCAAGGGGCTGAAAAG
	Reverse	5'GGAAGTTCCCAGAAGAGGTCA
*TNF*α	Forward	5'CAGCCTCTTCTCCTTCCTGAT
	Reverse	5'GCCAGAGGGCTGATTAGAGA
*CDKN2A*	Forward	5'CTCGTGCTGATGCTACTGAGGA
	Reverse	5'GGTCGGCGCAGTTGGGCTCC
*RGN*	Forward	5'AGGAAGTGTCCAACTCTCTGCT
	Reverse	5'TCTTGTCGTTATCCACCGTG
*CASP3*	Forward	5'GGAAGCGAATCAATGGACTCTGG
	Reverse	5'GCATCGACATCTGTACCAGACC

### 5.13 Immunofluorescent Staining

Fixed tissue sections (n ≥ 3), prepared as detailed in section 5.4 **Histology**, or scaffolds (N = 5), were permeabilized with 0.1% Triton for 10 min followed by PBS washes (3 × 10 min) (Merck). Samples were blocked for 2 h with 5% donkey serum, 5% bovine albumin serum (BSA, Sigma–Aldrich) in 0.01% PBS‐Tween‐20 (PBST, Sigma–Aldrich) at RT. For tissue samples, mouse‐on‐mouse IgG blocking reagent (Vector Laboratories) was added to negate non‐specific binding. Tissue sections utilized for Mφ staining were permeabilized with –20 °C acetone:methanol (1:1) for 1 min before being allowed to air dry and blocked with 1% BSA and FC block (1:500, Biolegend). All consecutive washes for Mφ staining were conducted using PBS. All samples were incubated with primary antibodies overnight at RT in 0.01% PBST (Table 4), followed by PBST or PBS washes (3 x 10 min). Antibodies were detected using Cy3‐, AlexaFluor 488‐ and AlexaFluor 647‐conjugated secondary Fab fragment antibodies (1:300; Jackson ImmunoResearch) and nuclei were stained using DAPI (1:1000, Sigma–Aldrich) in 0.01% PBST for 2 h at RT, while protected from light. Tissue sections were mounted using Prolong Gold anti‐fade mounting medium (Invitrogen) and scaffolds using 0.01% PBST. Images of tissue samples and scaffolds were acquired on a Leica SP8 4D and Zeiss LSM 980 Airyscan confocal microscope, respectively. Images of scaffolds seeded with non‐IR cells in standard culture conditions were obtained with a Nikon Ti‐E inverted wide‐field microscope. Fluorescent images were segmented using QuPath‐0.5.1 to identify individual cells by nuclei as regions of interest (ROIs). ROIs were counted and, if applicable, their relative mean fluorescent intensity (MFI) measured in ImageJ. As PCL diffracts fluorescent light, background fluorescence was removed from images containing cell‐seeded scaffolds post‐quantification for presentation purposes only.

**TABLE 4:** Antibodies for Immunofluorescent Staining.

Primary antibody	Dilution	Supplier	CAT#
Rat anti‐CD11b	1:200	Abcam	ab8878
Mouse anti‐ZO‐1	1:100	Invitrogen	33‐9100
Mouse anti‐TPO	1:100	Santa Cruz	sc‐374045
Mouse anti‐NKX2‐1	1:100	Invitrogen	8G7G3/1
Rabbit anti‐Tg	1:250	Abcam	EPR9730
Mouse anti‐T4	1:100	Santa Cruz	sc‐52247
Rat anti‐ECAD	1:400	Invitrogen	13‐1900
Mouse anti‐αSMA	1:400	Sigma	C6198
Rabbit anti‐KRT8	1:200	Sigma	SAB4501653
Mouse anti‐Ki67	1:200	BD Pharmingen	550609
Rabbit anti‐IBA1	1:200	Antibodies Online	ABIN2857032
Mouse anti‐CD206	1:200	Santa Cruz	sc‐58986
Mouse anti‐CD64	1:200	Santa Cruz	sc‐28347
Mouse anti‐CD86	1:200	Santa Cruz	sc‐28347
Rat anti‐CD163	1:800	Thermo Fisher	14‐1631‐82
Rabbit anti‐CSF1R	1:500	Cell Signalling Tech	43390s
Hamster anti‐CD11b	1:100	Biolegend	117302

### 5.14 Statistical Analysis

For all boxplots individual datapoints are indicated where possible and: box = interquartile range and median, whiskers = 5th–95th percentile. All other data was expressed as mean ± standard deviation. A Shapiro‐Wilks test was applied to ensure normal distribution, with *p ≥ 0.05 indicating that data was normally distributed. An F‐test was applied to determine equality of variance, with *p ≥ 0.05 indicating no significant difference in variance within the dataset. Significant results were determined using a t‐test or two‐way ANOVA with a *post‐hoc* Tukey test, as indicated, where *p ≤ 0.05, **p ≤ 0.1 and ***p ≤ 0.001. Statistical analysis was conducted using Minitab22 for all in vivo and in vitro work, and OriginLab version 2021b for all material characterization.

## Conflicts of Interest

The authors declare no conflicts of interest.

## Supporting information


**Supporting File**: adhm70798‐sup‐0001‐SuppMat.pdf.

## Data Availability

The data that support the findings of this study are available from the corresponding author upon reasonable request.
