# Supplementary material for: Targeting Post‐Irradiation Thyroid Dysfunction: Electrospun Scaffolds As A Dual‐Action Approach for Antioxidant and Immune Modulation
Source: Adv Healthc Mater. 2026 Feb 3;15(27):e01857. doi: 10.1002/adhm.202501857 (PMC13378484; doi:10.1002/adhm.202501857)
Supplement: Supplementary file 1 — Supporting File: adhm70798‐sup‐0001‐SuppMat.pdf. [file ADHM-15-0-s001.pdf]

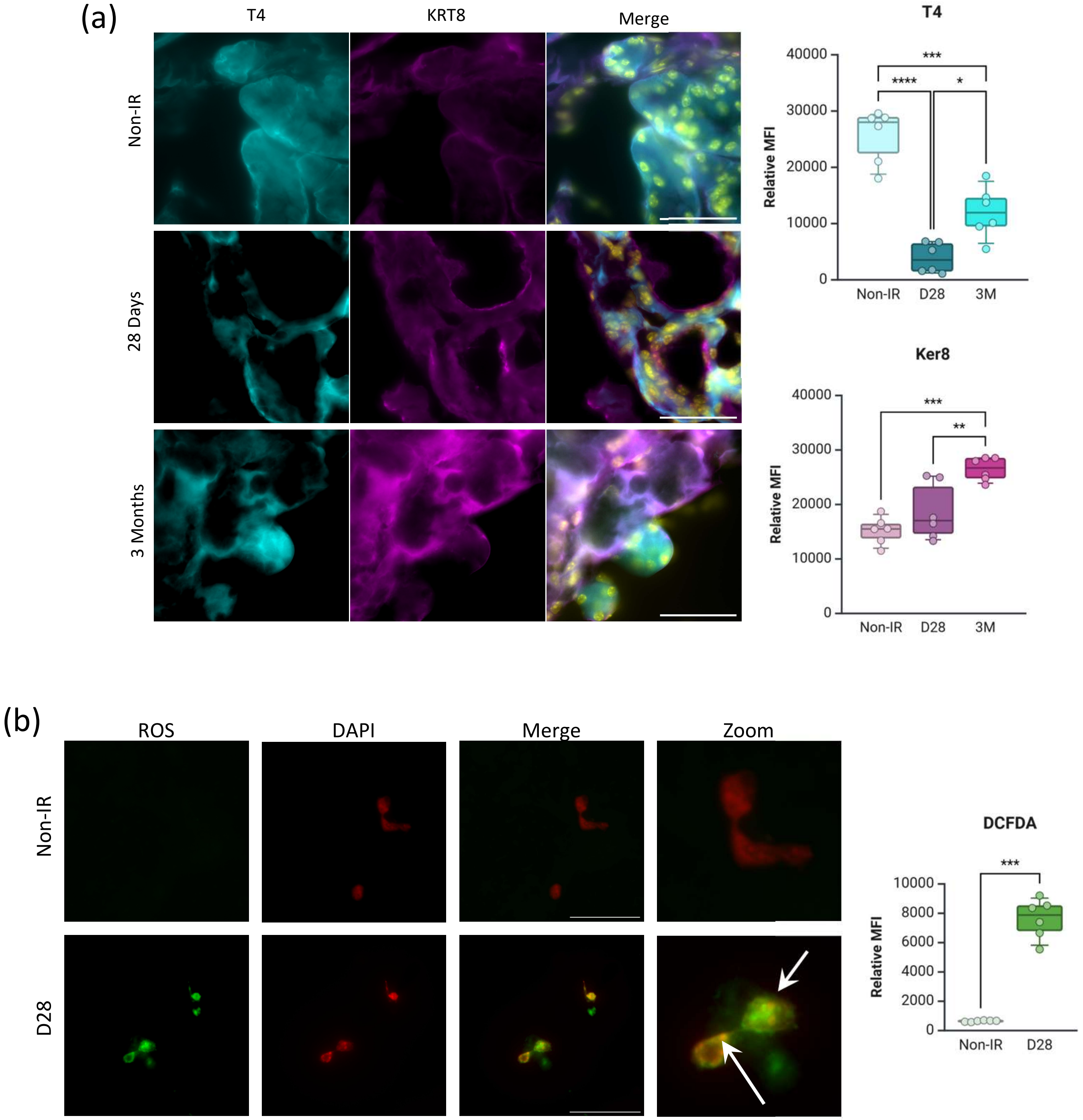

Supplementary Figure 1. – (a) Thyroid gland sections of mice pre- and 28 days post, and 3 months post-irradiation, immunofluorescently stained thyroxine (T4, cyan), Keratin 8 (KRT8, magenta) and DAPI (yellow). Relative mean fluorescent intensity (MFI) quantification is displayed on the right. Statistical analysis: one-way ANOVA and post hoc Tukey. (b) DCFDA staining indicating ROS (green) of thyroid gland single-cell suspensions obtained from mice pre- and 28 days post-irradiation, counterstained with DAPI (red). White arrows indicate localization of ROS in the nuclei. Relative mean fluorescent intensity (MFI) quantification is displayed on the right. Statistical analysis: Welch's t-test *p≤0.05, **p ≤ 0.01, ***p ≤ 0.001, ****p ≤ 0.0001. Box = interquartile range and median, whiskers = 5th-95th percentile. N=6. Scale bars = 60µm, x60 magnification

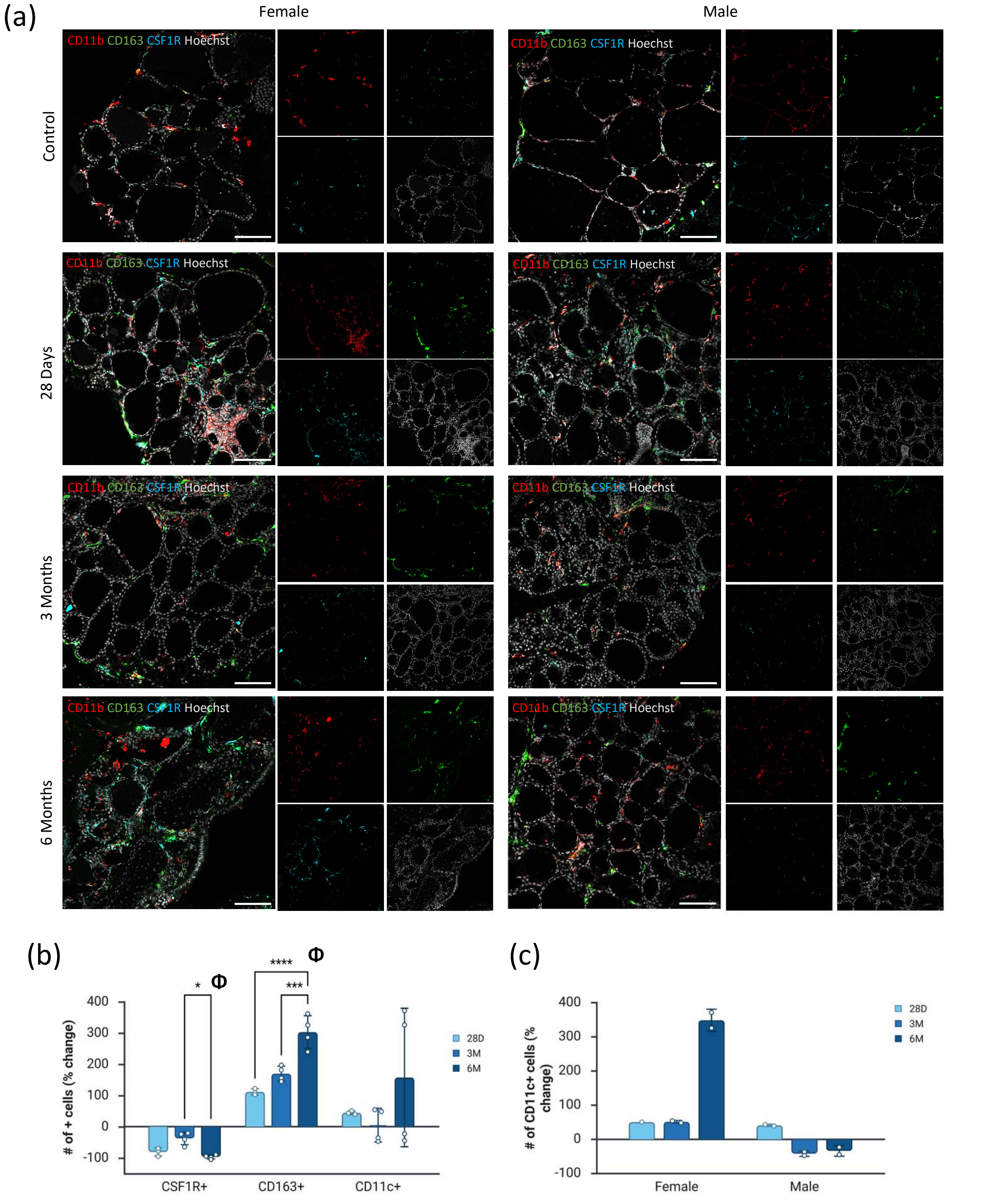

Supplementary Figure 2. – (a) Thyroid gland sections of female (right) and male (left) mice pre- and 28 days post-, 3 months post- and 6 months post-irradiation, immunofluorescently stained for CD11b (red), CD163 (green), CSF1R (cyan) and Hoechst (grey). Scale bars = 60µm, x60 magnification (b) The % change of the number of positive cells in comparison to the sex-specific control mean. Statistical analysis: one-way ANOVA and post hoc Bonferroni. $*p \leq 0.05$, $**p \leq 0.01$, $***p \leq 0.001$, $****p \leq 0.0001$. Data = mean ± SD. N≥3. (c) The % change of CD11c+ cells for each sex.

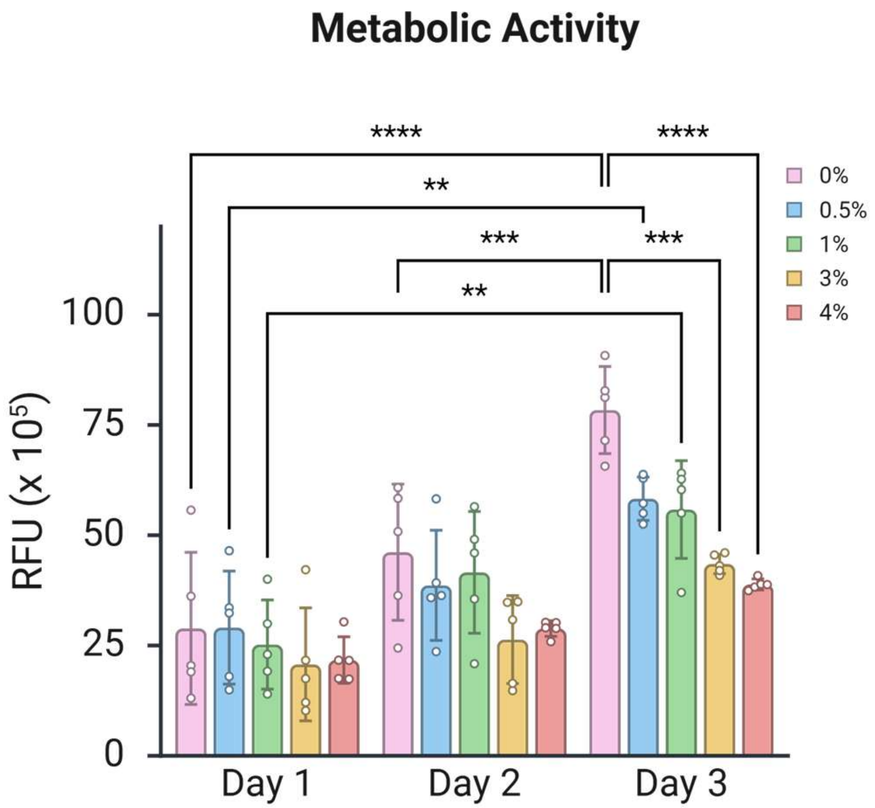

Supplementary Figure 3. – Metabolic activity of Nthy-ori 3-1 cells in the presence of various concentrations of free adenosine blended in media. Statistical analysis: two-way ANOVA and post hoc Tukey. *p≤0.05, **p ≤ 0.01, ***p ≤ 0.001, ****p ≤ 0.0001. Data = mean ± SD. N=5

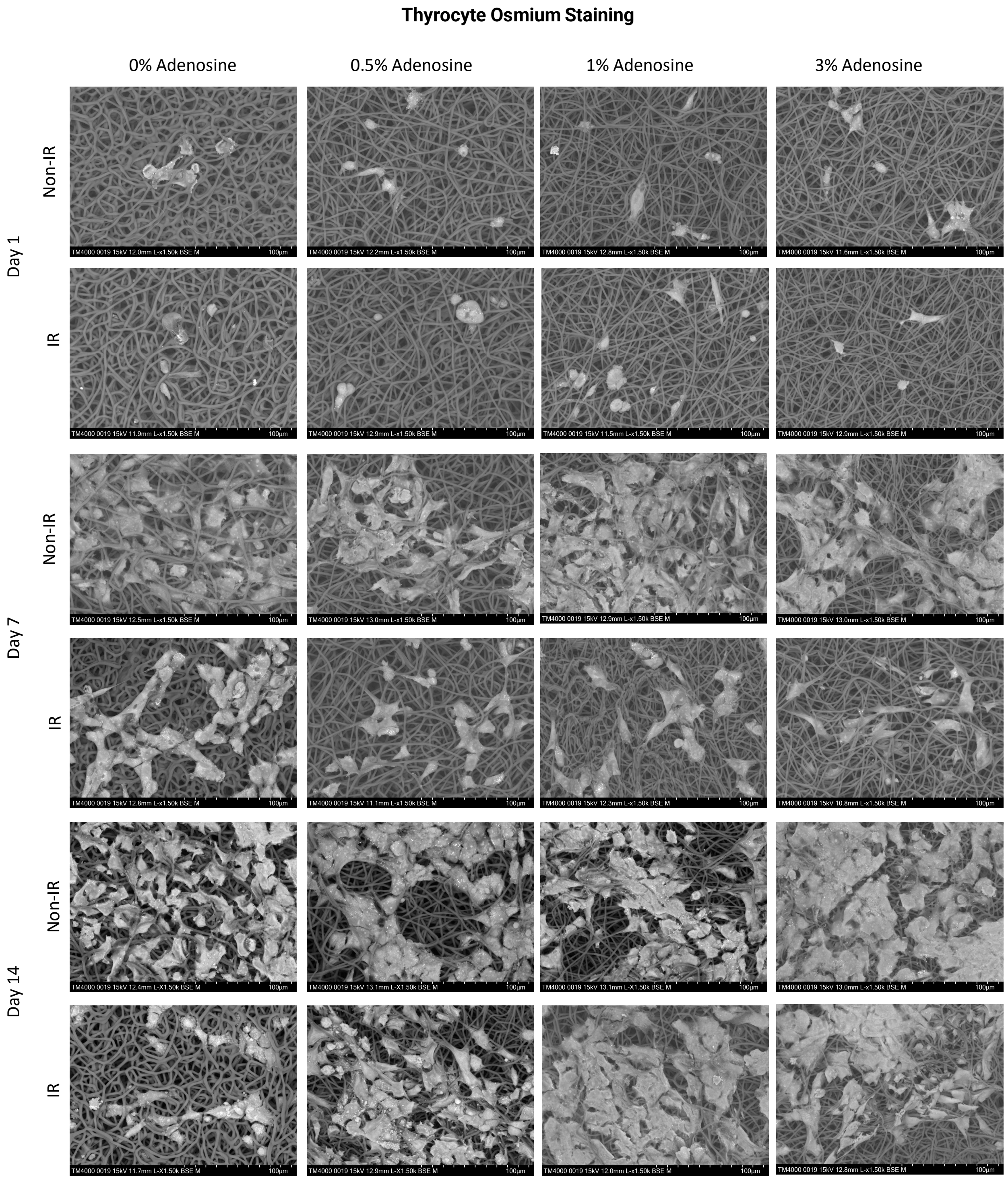

Supplementary Figure 4. - SEM images of electrospun PCL-only and 0.5, 1 and 3% adenosine scaffolds seeded with non-irradiated (Non-IR) or irradiated (IR) Nthy-ori 3-1 cells and stained with osmium at day 1, 7 and 14 of culture. scale bar=100μm, x1500 magnification.

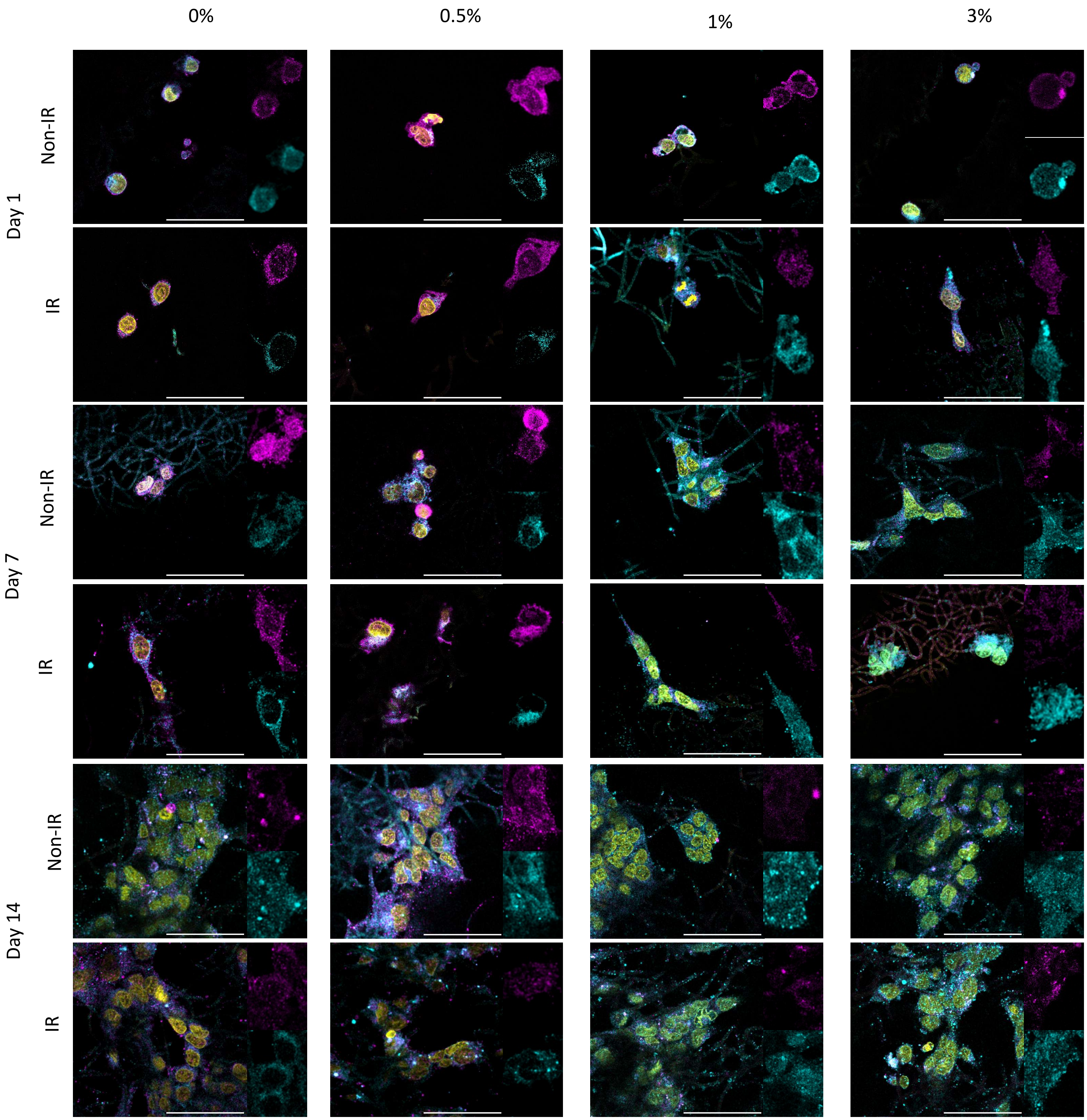

Supplementary Figure 5. - Images of electrospun PCL-only and 0.5, 1 and 3% adenosine scaffolds seeded with Nthy-ori 3-1 cells and immunofluorescently stained for E-Cadherin (ECAD, cyan), alpha smooth-muscle actin (αSMA, magenta) and DAPI (yellow) at days 1, 7 and 14 of culture. Scale bars = 60µm, x60 magnification.

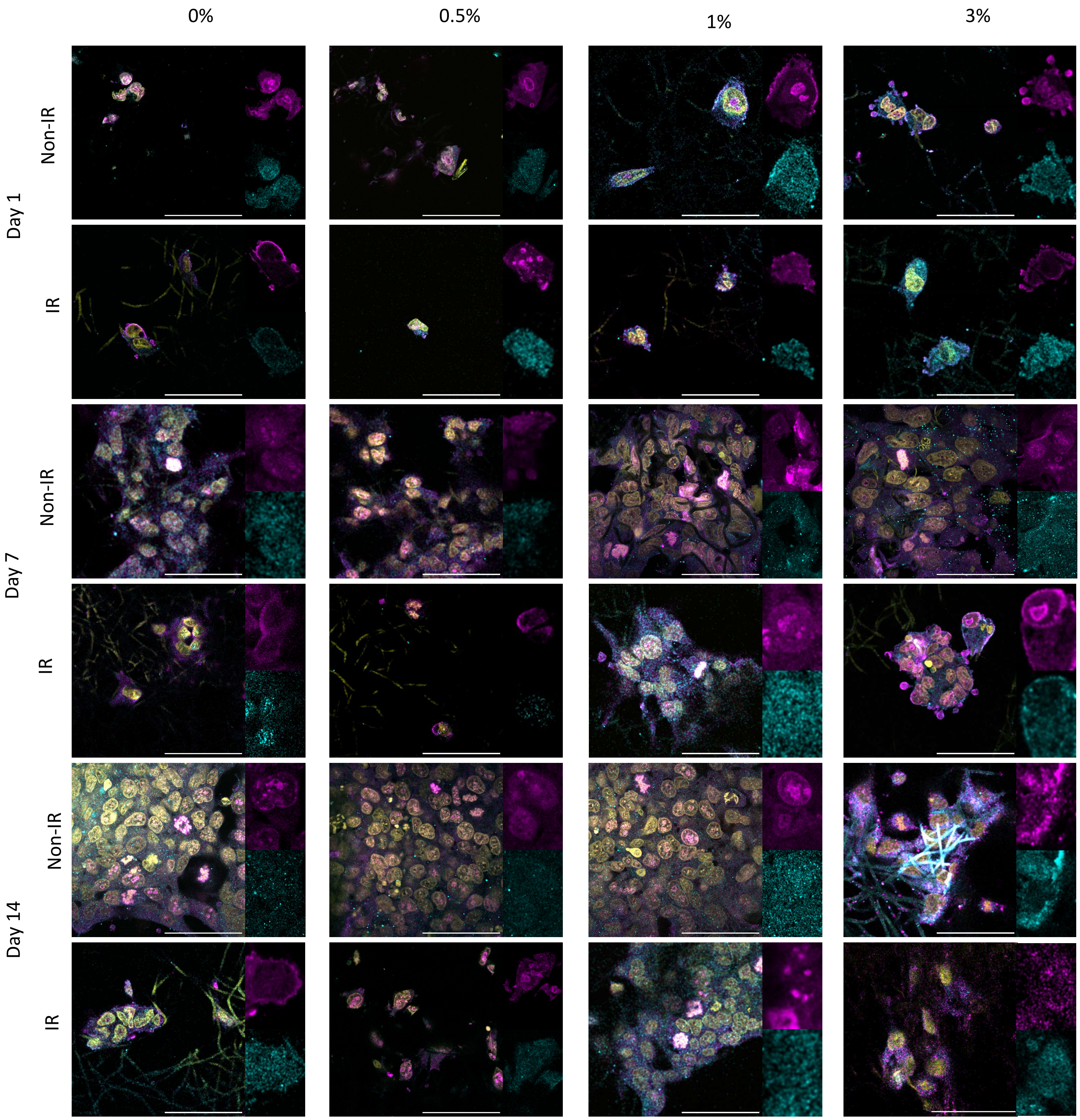

Supplementary Figure 6. - Images of electrospun PCL-only and 0.5, 1 and 3% adenosine scaffolds seeded with Nthy-ori 3-1 cells and immunofluorescently stained for Thyroglobulin (Tg, cyan), marker of proliferation Kiel 67 (ki67, magenta) and DAPI (yellow) at days 1, 7 and 14 of culture. Scale bars = 60µm, x60 magnification.

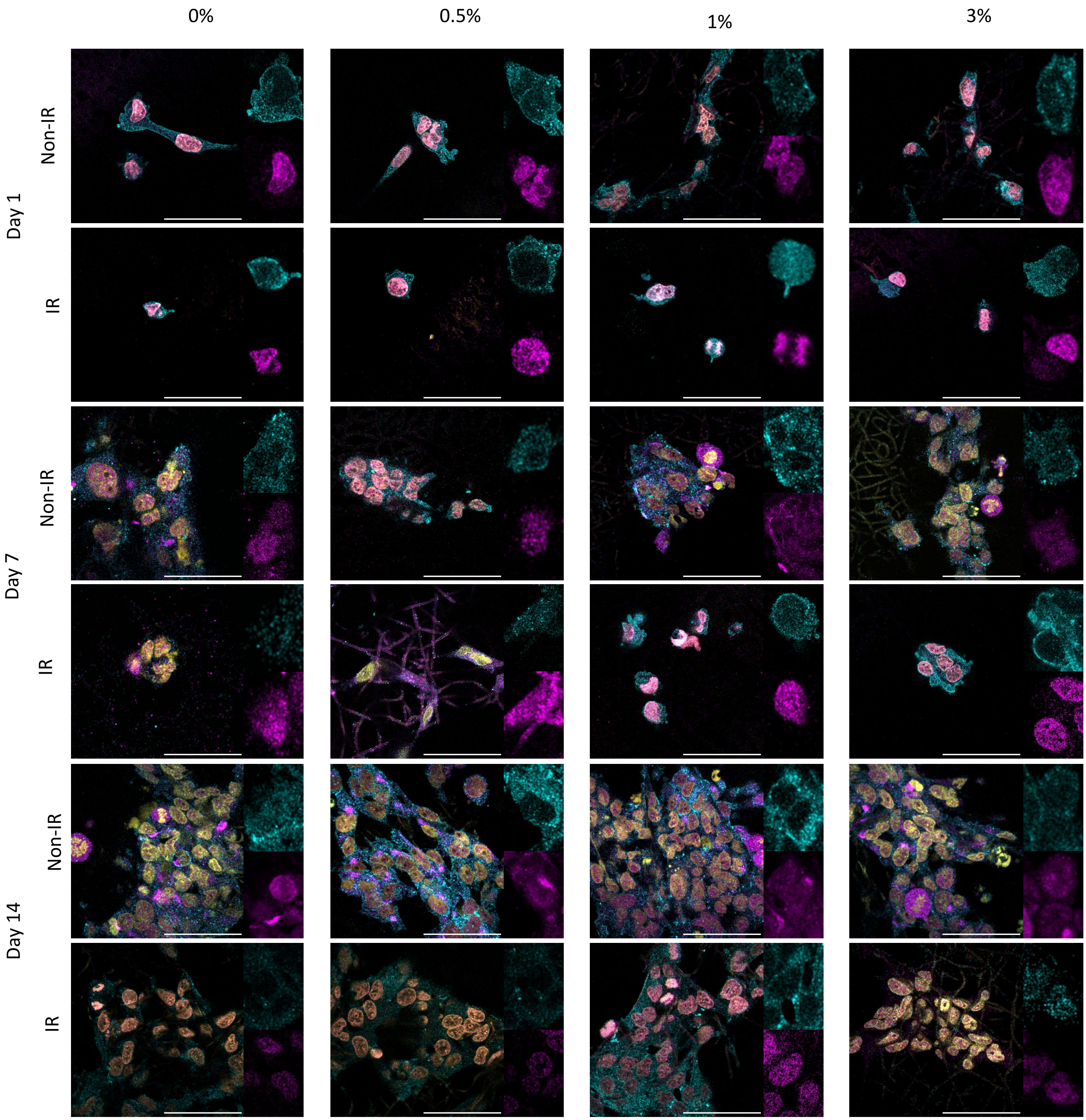

Supplementary Figure 7. - Images of electrospun PCL-only and 0.5, 1 and 3% adenosine scaffolds seeded with Nthy-ori 3-1 cells and immunofluorescently stained for Zonula Occludens 1 (ZO-1, magenta), Keratin 8 (KRT8, cyan) and DAPI (yellow) at days 1, 7 and 14 of culture. Scale bars = 60µm, x60 magnification.

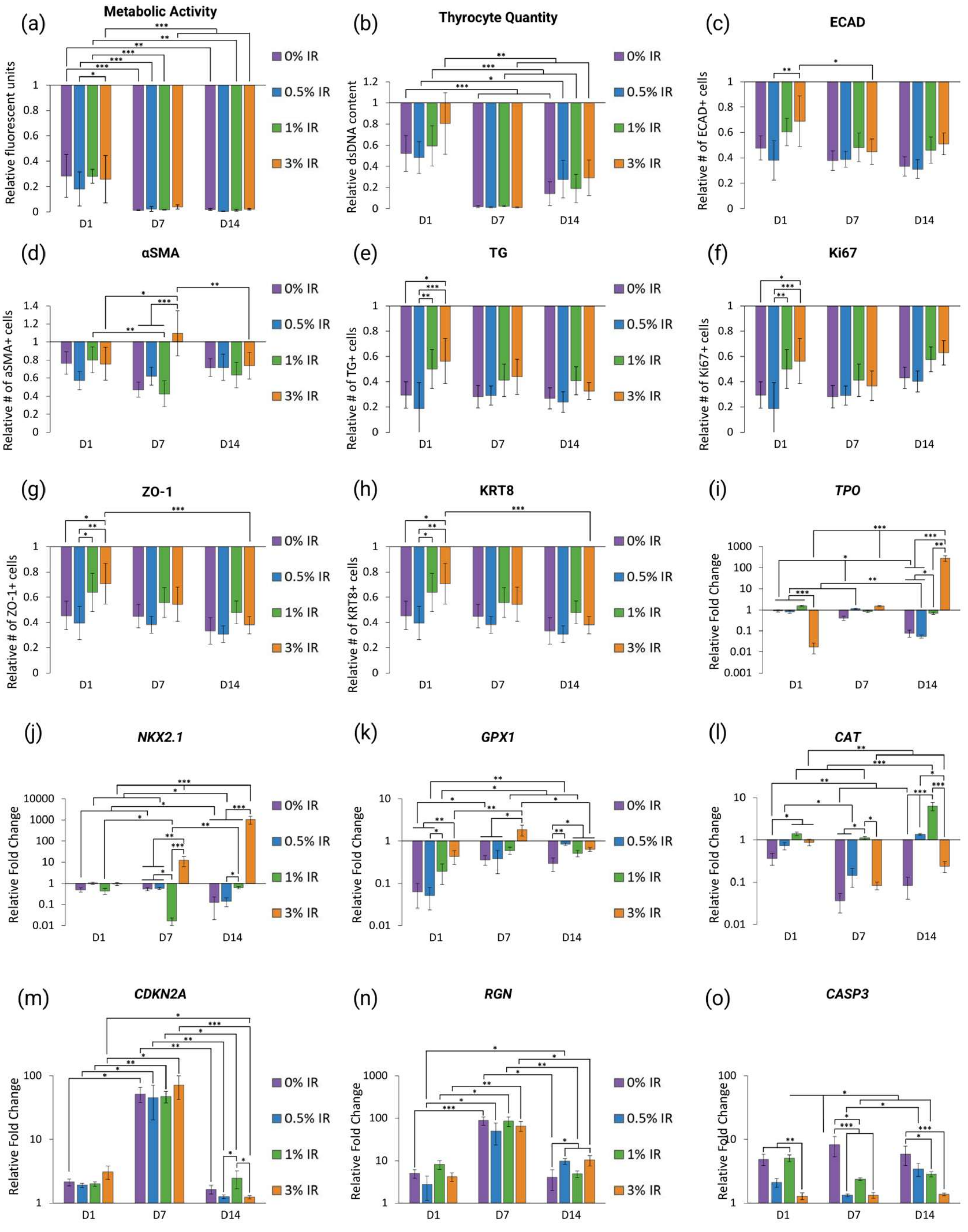

Supplementary Figure 8 - Responses of irradiated Nthy-ori 3-1 cells to electrospun adenosine scaffolds normalized tonon-irradiated cells on the same day cultured on the same adenosine % displaying: (a) metabolic activity, (b) dsDNAquantification, (c) E-Cadherin (ECAD), (d) alpha-smooth muscle actin (αSMA), (e) thyroglobulin (TG), (f) Antigen Kiel67 (Ki67), (g) Zonnula Occludens 1 (ZO-1), (h) cytokeratin 8 (KRT8), (i) thyroid peroxidase (TPO), (j) NK2 homeobox 1(NKX2-1), (k) glutathionine peroxidase 1 (GPX1), (l) catalase (CAT), (m) Cyclin-Dependent Kinase Inhibitor 2A (CDKN2A),(n) regucalcin (RGN) and (o) caspase 3 (CASP3). N=5. Statistical analysis: two-way ANOVA and post hoc Tukey, *p≤0.05,**p≤0.01, ***p≤0.001. Data = mean ± SD. Scale bars = 100μm

Macrophage Osmium Staining

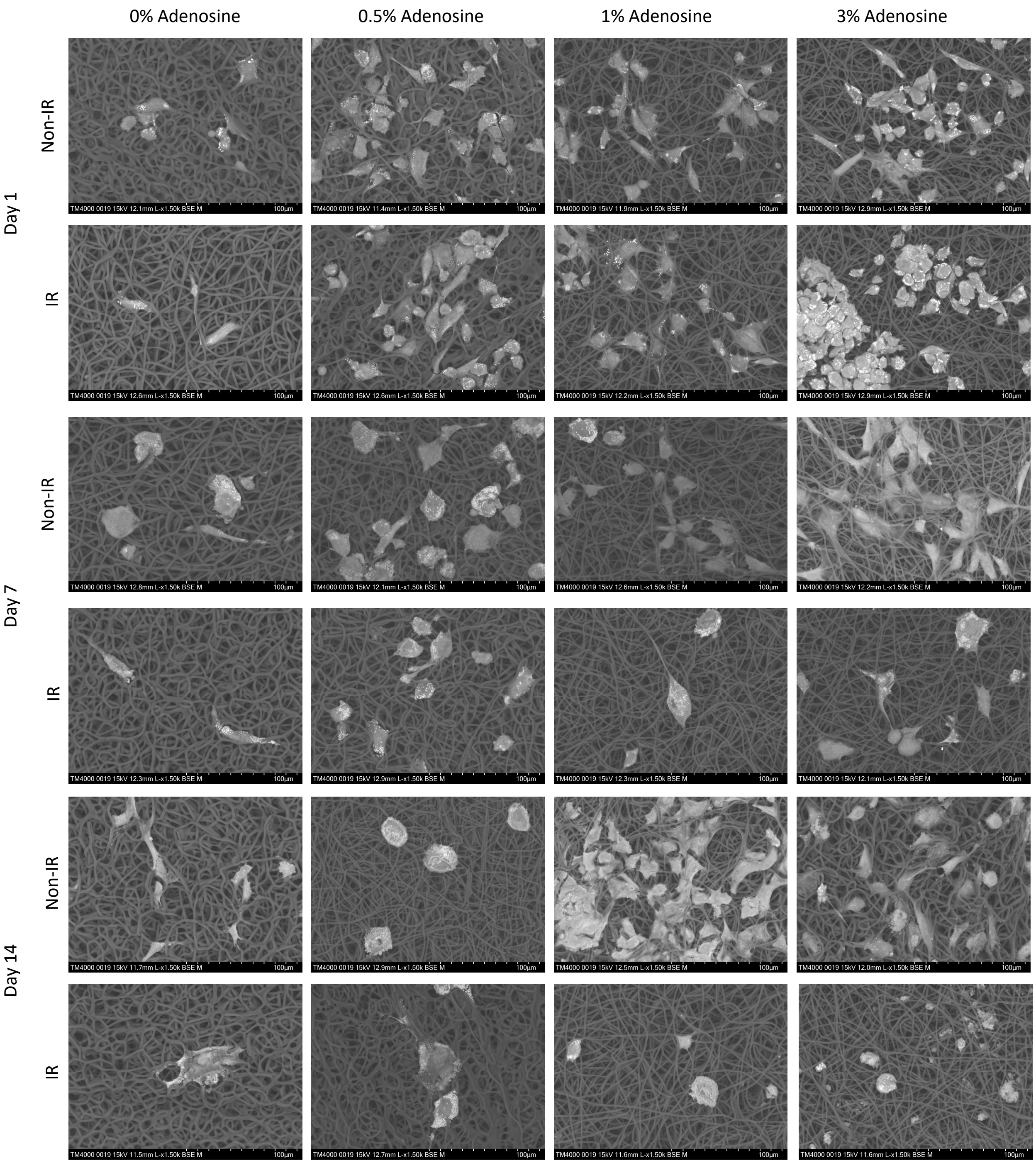

Supplementary Figure 9. - SEM images of electrospun PCL-only and 0.5, 1 and 3% adenosine scaffolds seeded with non-irradiated (Non-IR) or irradiated (IR) THP-1-derived macrophages and stained with osmium at day 1, 7 and 14 of culture. scale bar=100µm, x1500 magnification.

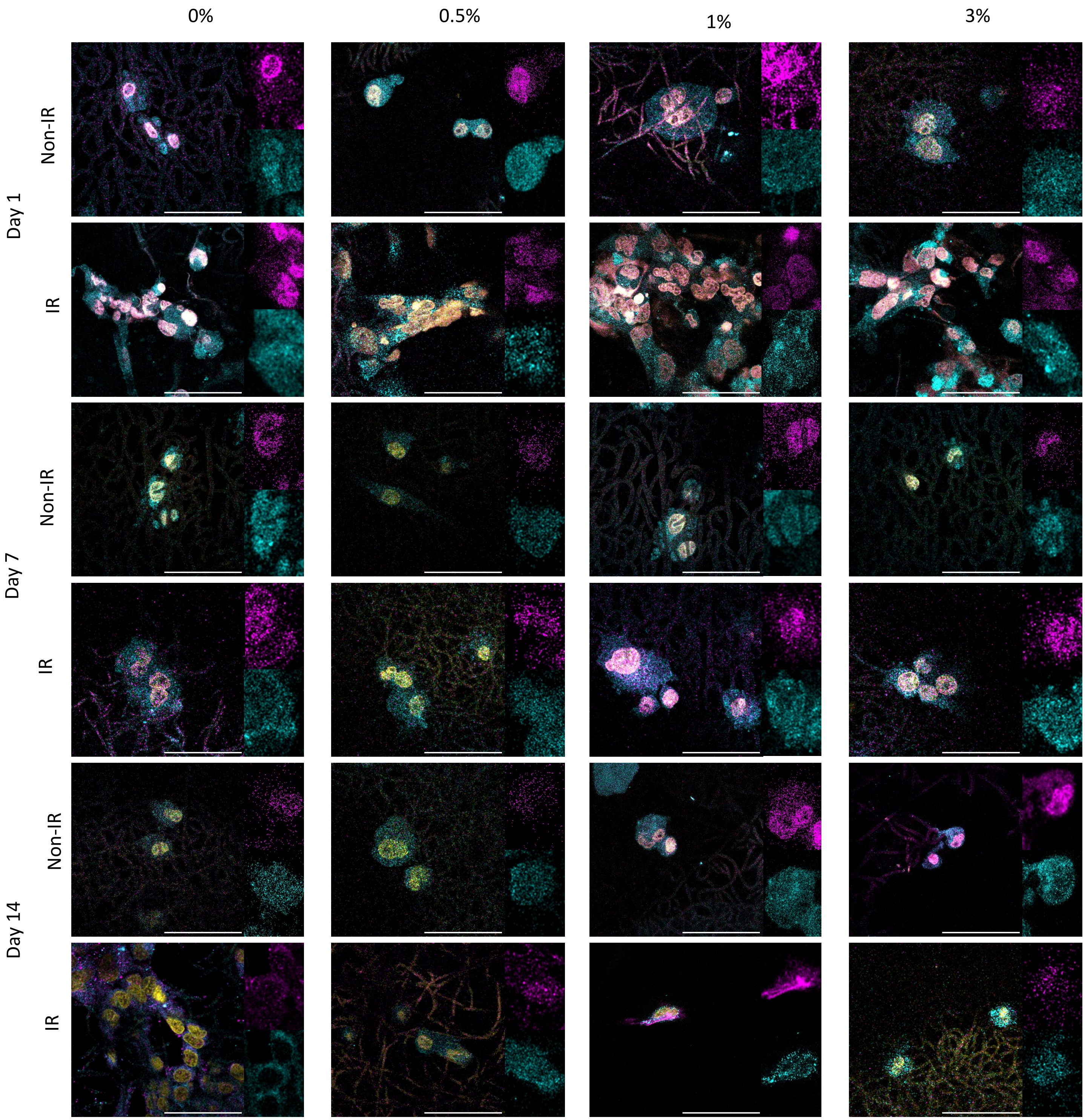

Supplementary Figure 10. - Images of electrospun PCL-only and 0.5, 1 and 3% adenosine scaffolds seeded with THP-1-derived macrophages and immunofluorescently stained CD11b (cyan), CD64 (magenta) and DAPI (yellow) at days 1, 7 and 14 of culture. Scale bars = 60µm, x60 magnification.

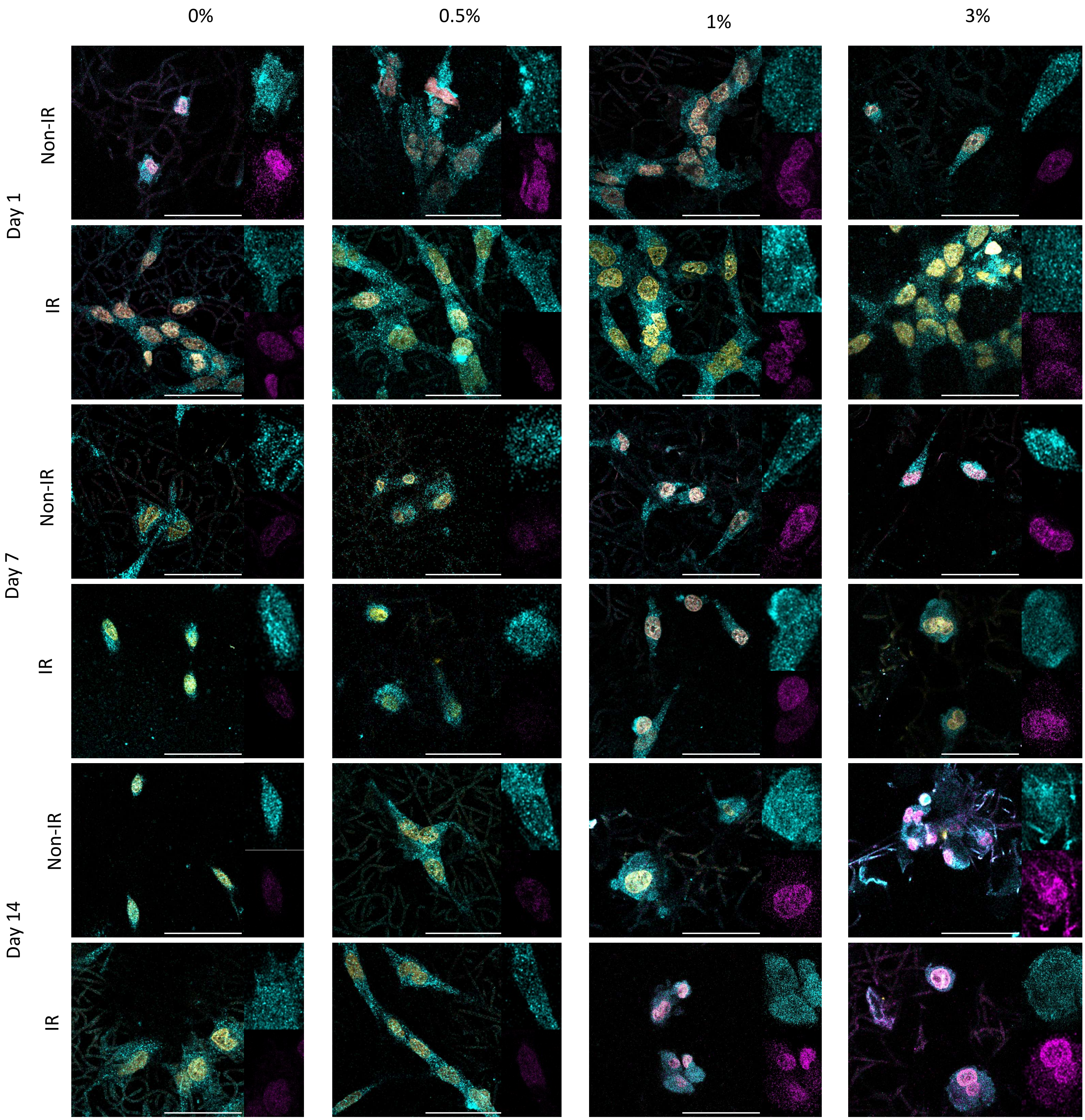

Supplementary Figure 11. - Images of electrospun PCL-only and 0.5, 1 and 3% adenosine scaffolds seeded with THP-1-derived macrophages and immunofluorescently stained for IBA1 (IBA1, cyan), CD206 (magenta) and DAPI (yellow) at days 1, 7 and 14 of culture. Scale bars = 60µm, x60 magnification.

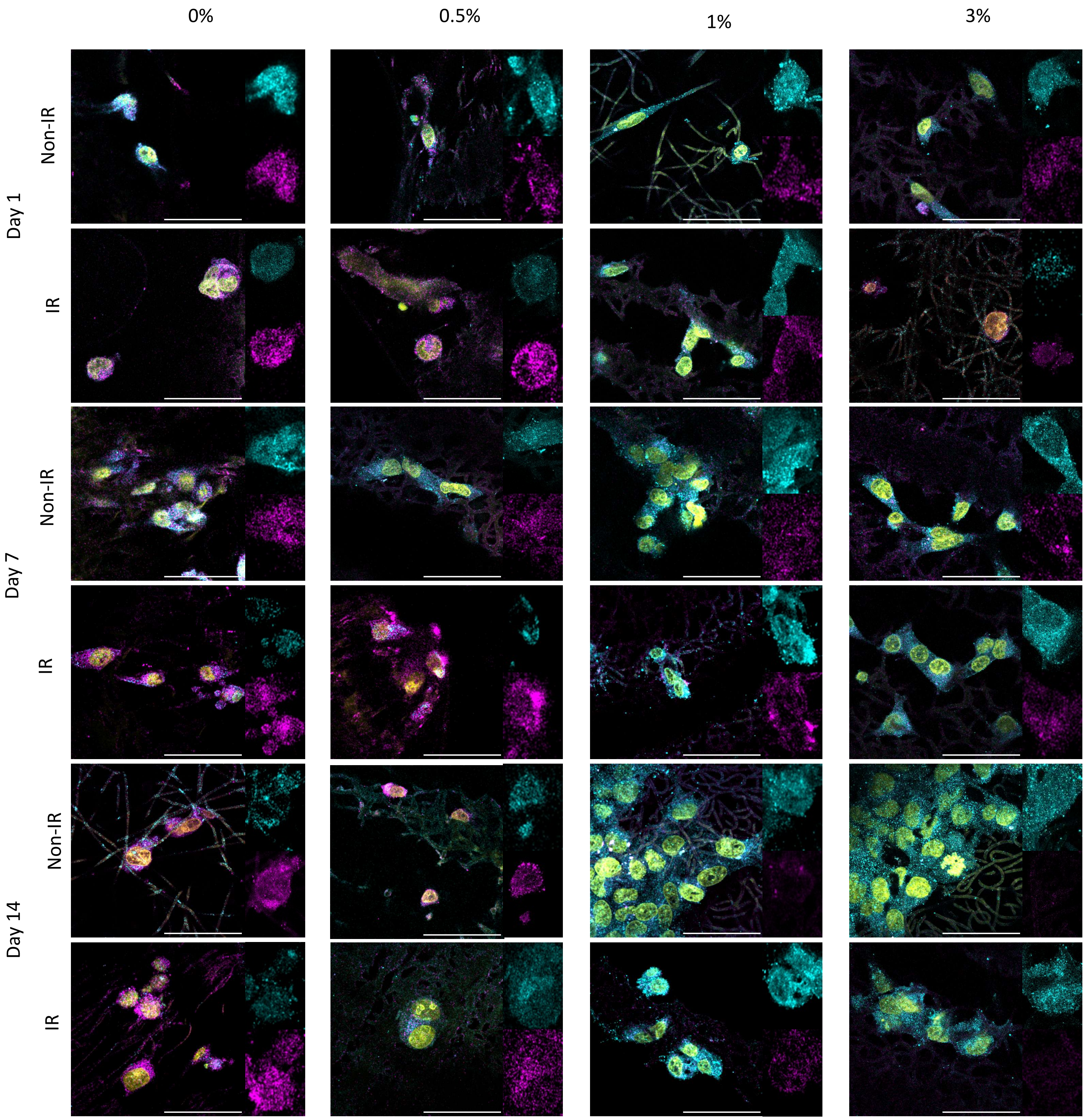

Supplementary Figure 12. - Images of electrospun PCL-only and 0.5, 1 and 3% adenosine scaffolds seeded with THP-1-derived macrophages and immunofluorescently stained for CD163 (cyan), CD86 (magenta) and DAPI (yellow) at days 1, 7 and 14 of culture. Scale bars = 60µm, x60 magnification.

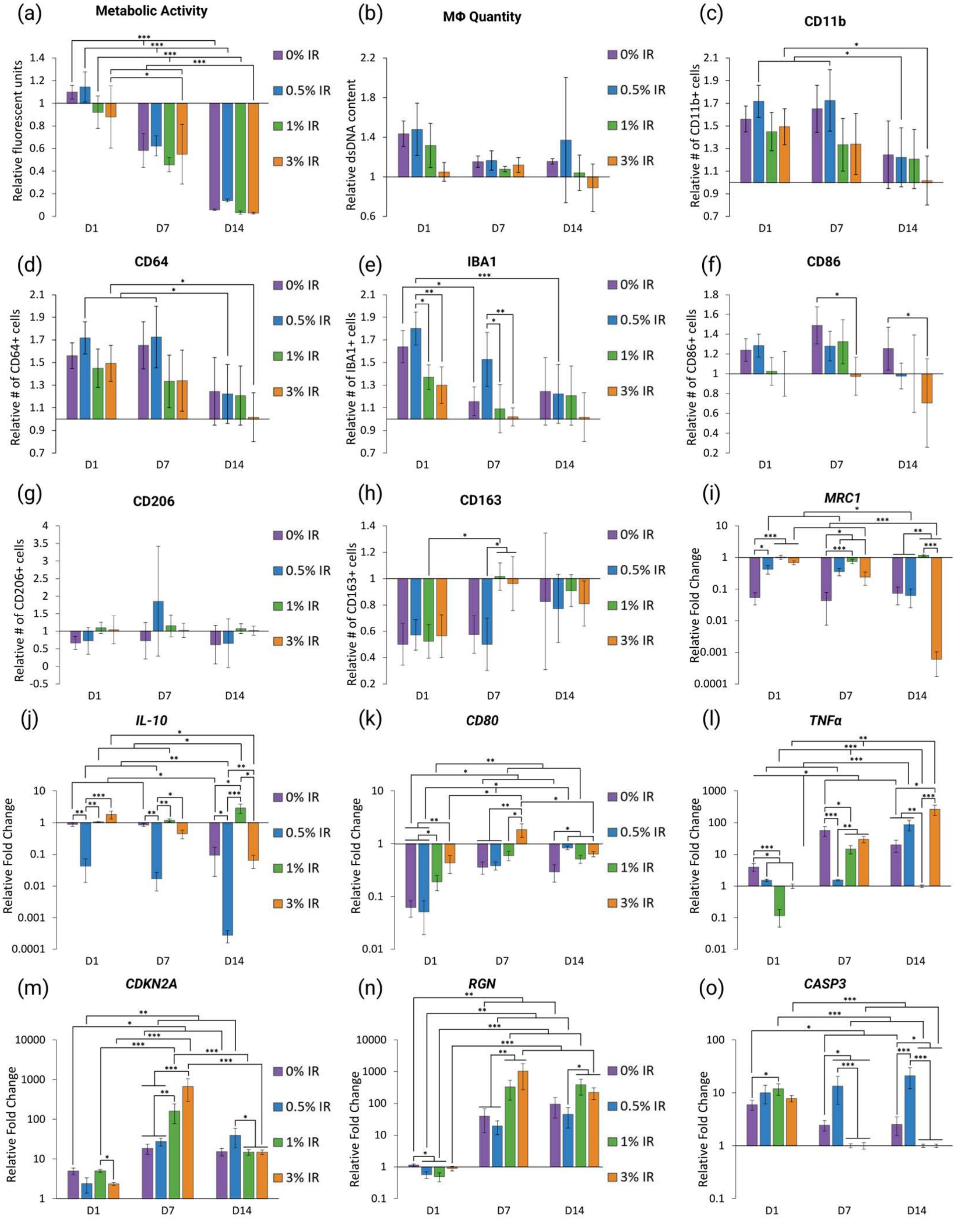

Supplementary Figure 13. - Responses of irradiated THP-1-derived macrophages (Mϕ) to electrospun adenosine scaffoldsnormalized to non-irradiated cells on the same day cultured on the same adenosine % displaying: (a) metabolic activity, (b)dsDNA quantification, (c) CD11b, (d) CD64, (e) ionized calcium-binding adapter molecule 1 (IBA1), (f) CD86, (g) CD206, (h)CD163, (i) mannose receptor C-type 1 (MRC1), (j) interleukin 10 (IL-10), (k) CD80, (l) tumor-necrosis factor alpha (TNFα),(m) Cyclin-Dependent Kinase Inhibitor 2A (CDKN2A), (n) regucalcin (RGN) and (o) caspase 3 (CASP3). N=5. Statisticalanalysis: two-way ANOVA and post hoc Tukey, *p≤0.05, **p≤0.01, ***p≤0.001. Data = mean ± SD. Scale bars = 100μm

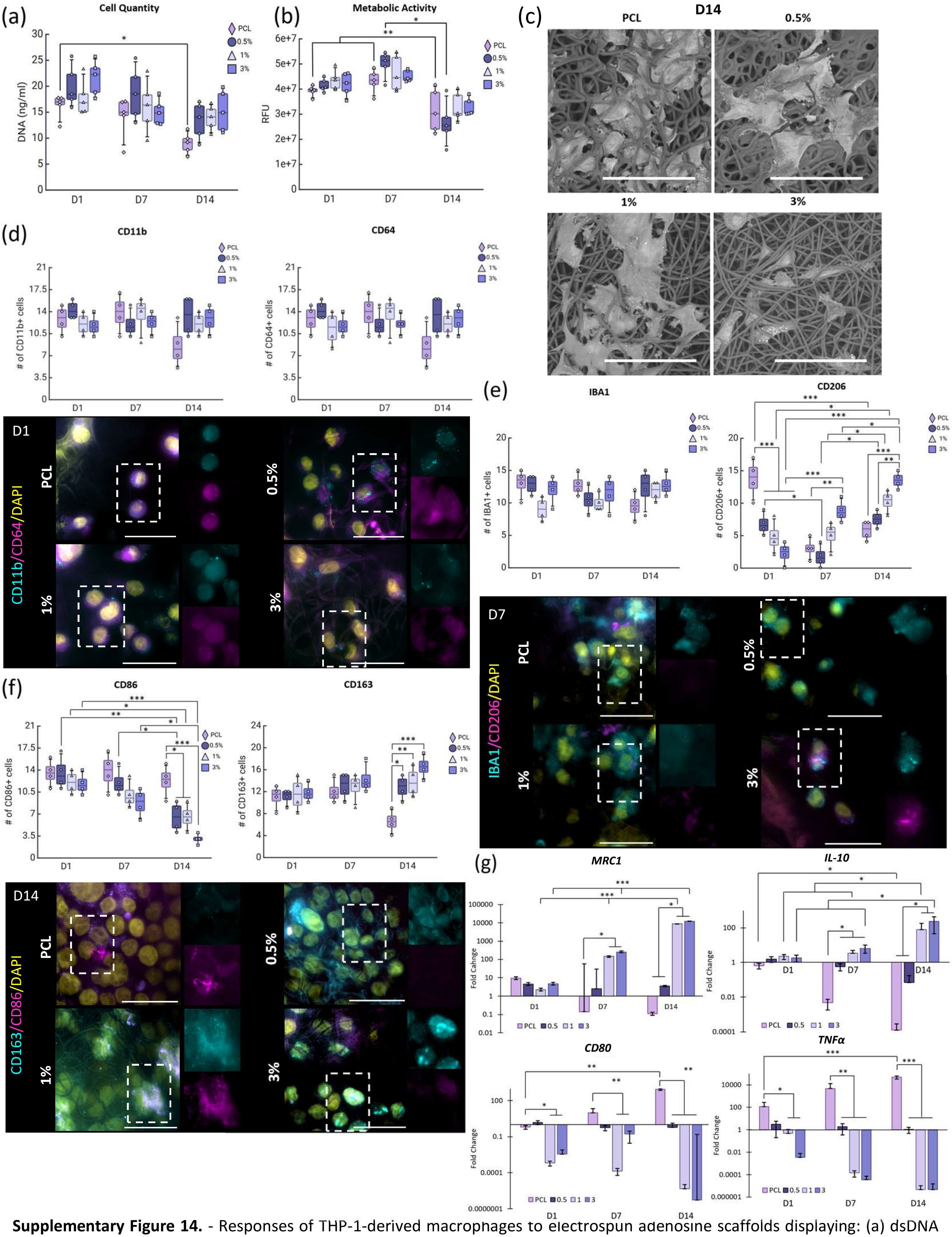

Supplementary Figure 14. - Responses of THP-1-derived macrophages to electrospun adenosine scaffolds displaying: (a) dsDNA quantification, (b) metabolic activity and (c) representative osmium-stained SEM at day 14. IF images of cell-seeded scaffolds stained for (d) CD11b (cyan) and CD64 (magenta), (e) CD206 (magenta) and ionized calcium-binding adapter molecule (IBA1, cyan), and (f) CD163 (cyan) and CD86 (magenta). (g) qRT-PCR analysis of macrophage plasticity-associated gene expression including MRC1 (encoding CD206), interleukin 10 (IL-10), CD80 and tumour necrosis factor alpha (TNFα). N=5. Statistical analysis: two-way ANOVA and post hoc Tukey, *p>0.05, **p>0.01, ***p>0.001. Box = interquartile range and median, whiskers = 5th-95th percentile. Scale bars = 40μm, x60 magnification.

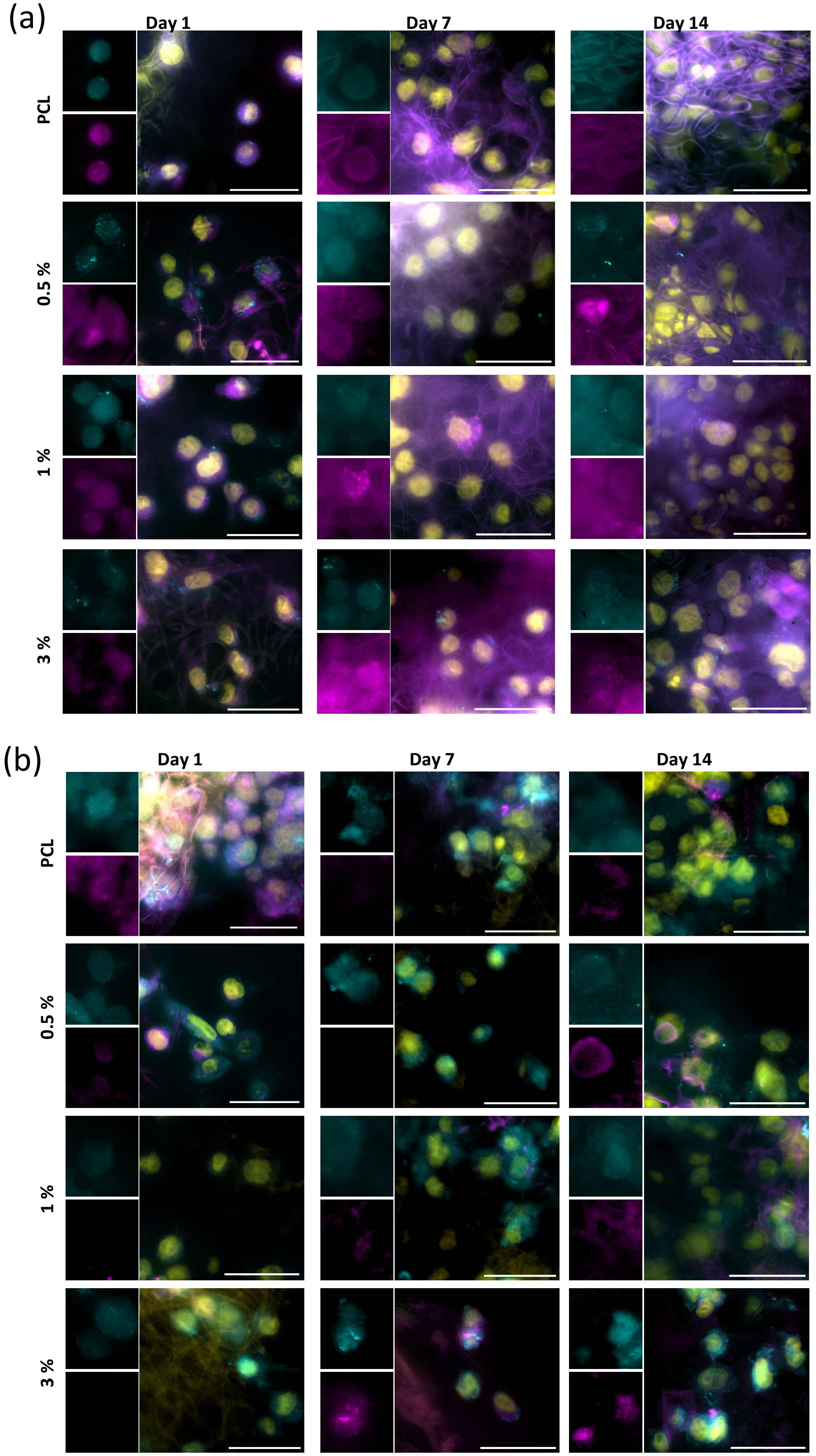

Supplementary Figure 15. – Images of electrospun PCL-only and 0.5, 1 and 3% adenosine scaffolds seeded with THP-1-derived macrophages and immunofluorescently stained for (a) CD11b (cyan), CD64 (magenta) and DAPI (yellow) and (b) stained for IBA1 (cyan), CD206 (magenta) and DAPI (yellow) at days 1, 7 and 14 of culture. Scale bars = 40µm, x60 magnification.

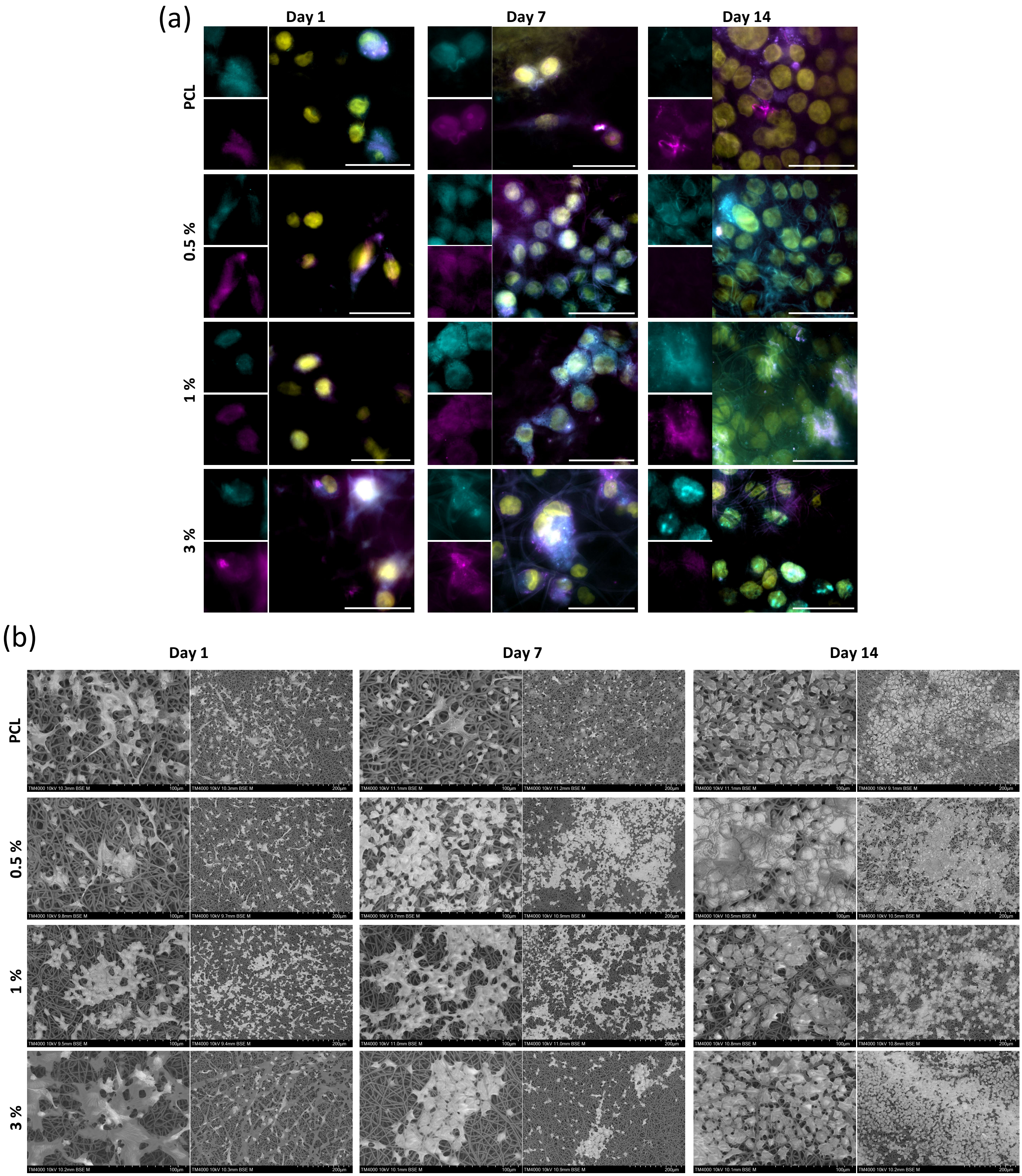

Supplementary Figure 16. – (a) Images of electrospun PCL-only and 0.5, 1 and 3% adenosine scaffolds seeded with THP-1-derived macrophages and immunofluorescently stained for CD163 (cyan), CD86 (magenta) and DAPI (yellow) at days 1, 7 and 14 of culture. Scale bars = 40µm, x60 magnification. (b) SEM images of electrospun PCL-only and 0.5, 1 and 3% adenosine scaffolds seeded with THP-1-derived macrophages and stained with osmium at day 1, 7 and 14 of culture. Left columns: scale bar=100µm, x1500 magnification. Right columns: scale bar=200µm, x500 magnification.

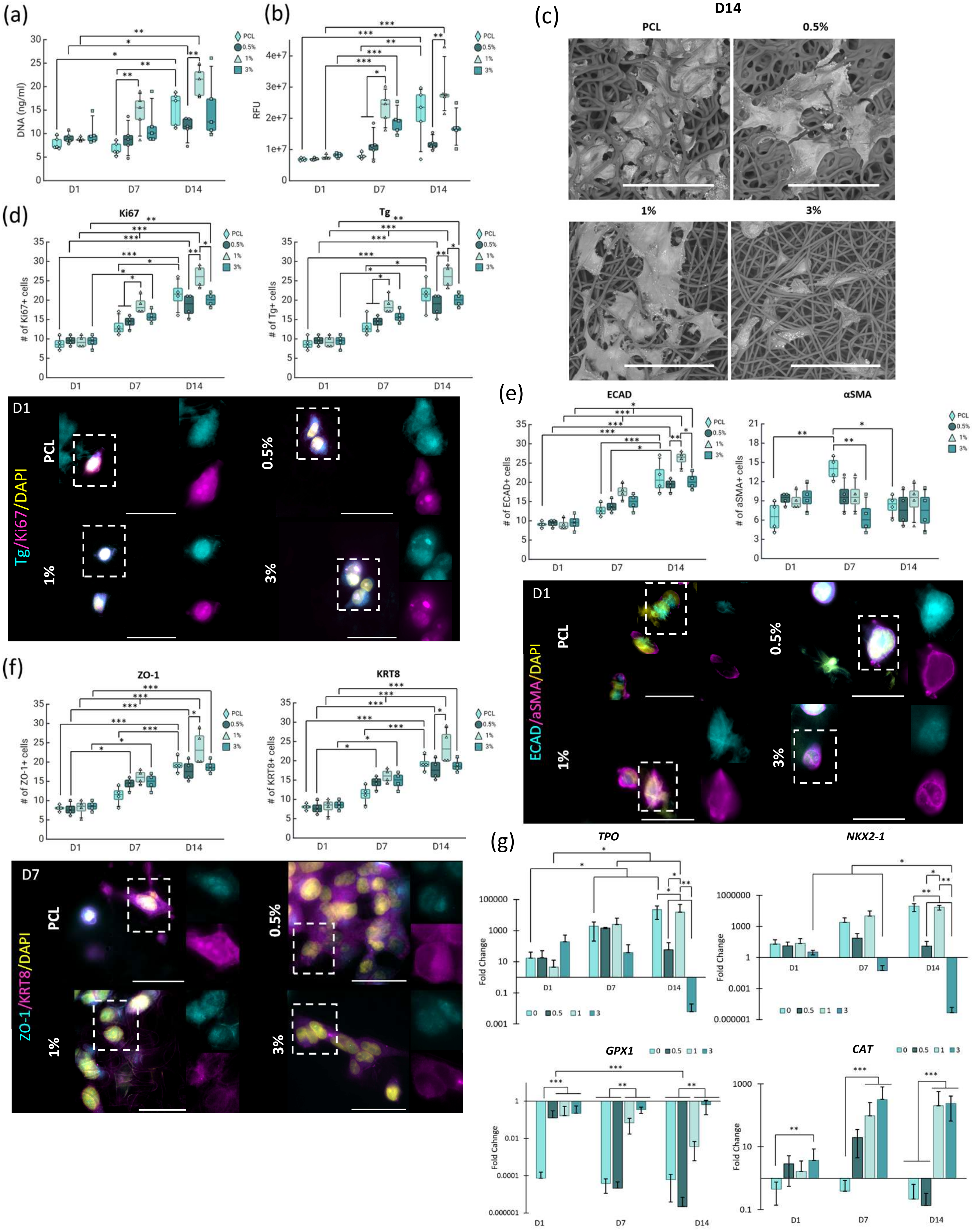

Supplementary Figure 17. - Responses of Nthy-ori 3-1 cells to electrospun adenosine scaffolds displaying: (a) dsDNA quantification, (b) metabolic activity and (c) representative osmium-stained SEM at day 14. IF images of cell-seeded scaffolds stained for (d) Thyroglobulin (Tg, cyan) and Antigen Kiel 67 (Ki67, magenta), (e) E-Cadherin (ECAD, cyan) and α-smooth-muscle actin (αSMA, cyan), and (f) Zonnula Occludens 1 (ZO-1, cyan) and cytokeratin 8 (KRT8, magenta). (g) qRT-PCR analysis of gene expression of thyroid-specific proteins thyroid peroxidase (TPO) and NK2 Homeobox 1 (NKX2.1), as well as antioxidant enzymes glutathione (GPx1) and catalase (CAT). N=5. Statistical analysis: two-way ANOVA and post hoc Tukey, *p>0.05, **p>0.01, ***p>0.001. Box = interquartile range and median, whiskers = 5th-95th percentile. Scale bars = 40μm, x60 magnification.

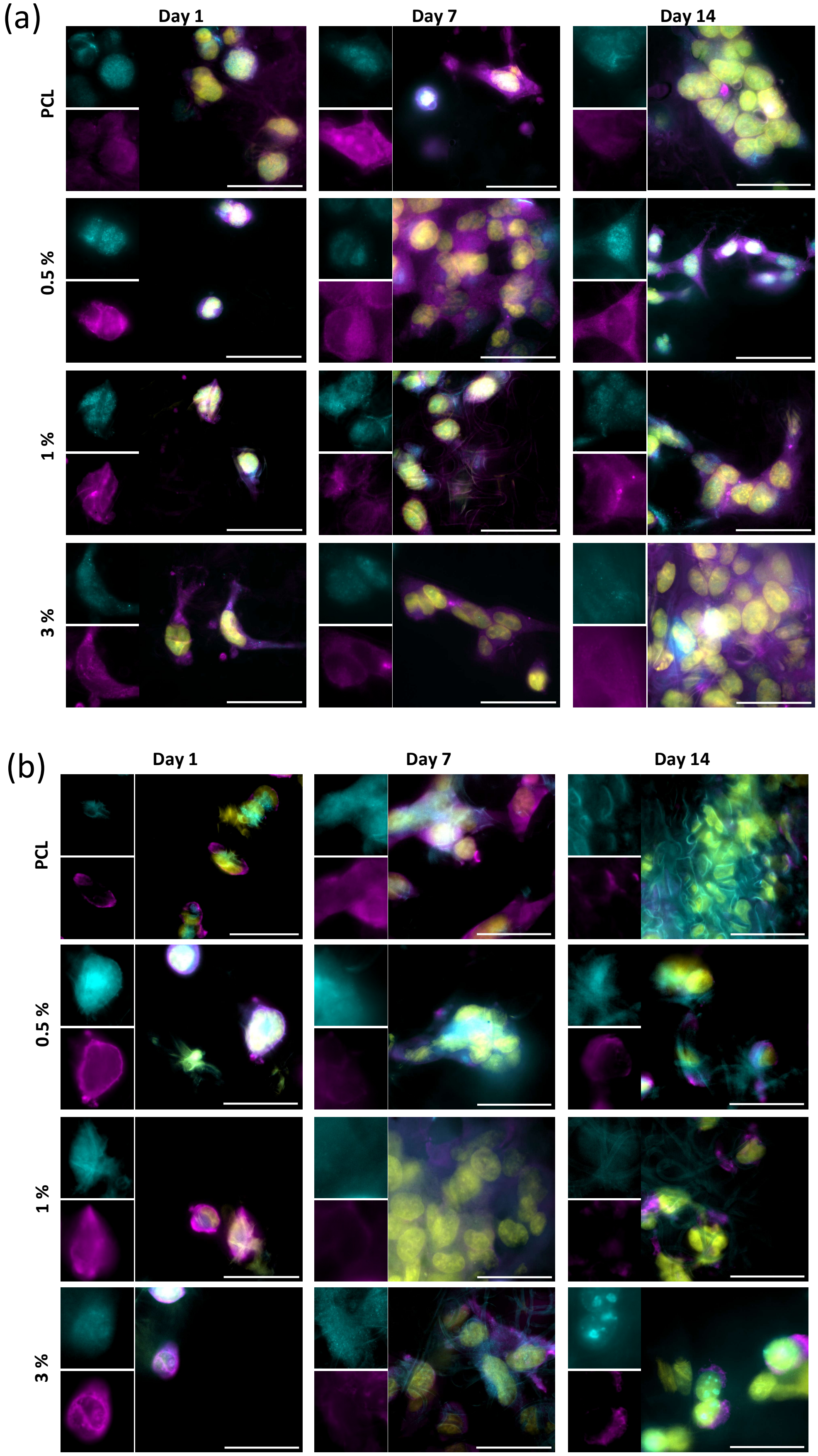

Supplementary Figure 18. – Images of electrospun PCL-only and 0.5, 1 and 3% adenosine scaffolds seeded with Nthy-ori 3-1 cells and immunofluorescently stained for (a) Keratin 8 (KRT8, cyan), Zonnula Occludens 1 (ZO-1, magenta) and DAPI (yellow) and (b) stained for E-Cadherin (ECAD, cyan), alpha smooth-muscle actin (αSMA, magenta) and DAPI (yellow) at days 1, 7 and 14 of culture. Scale bars = 40μm, x60 magnification.

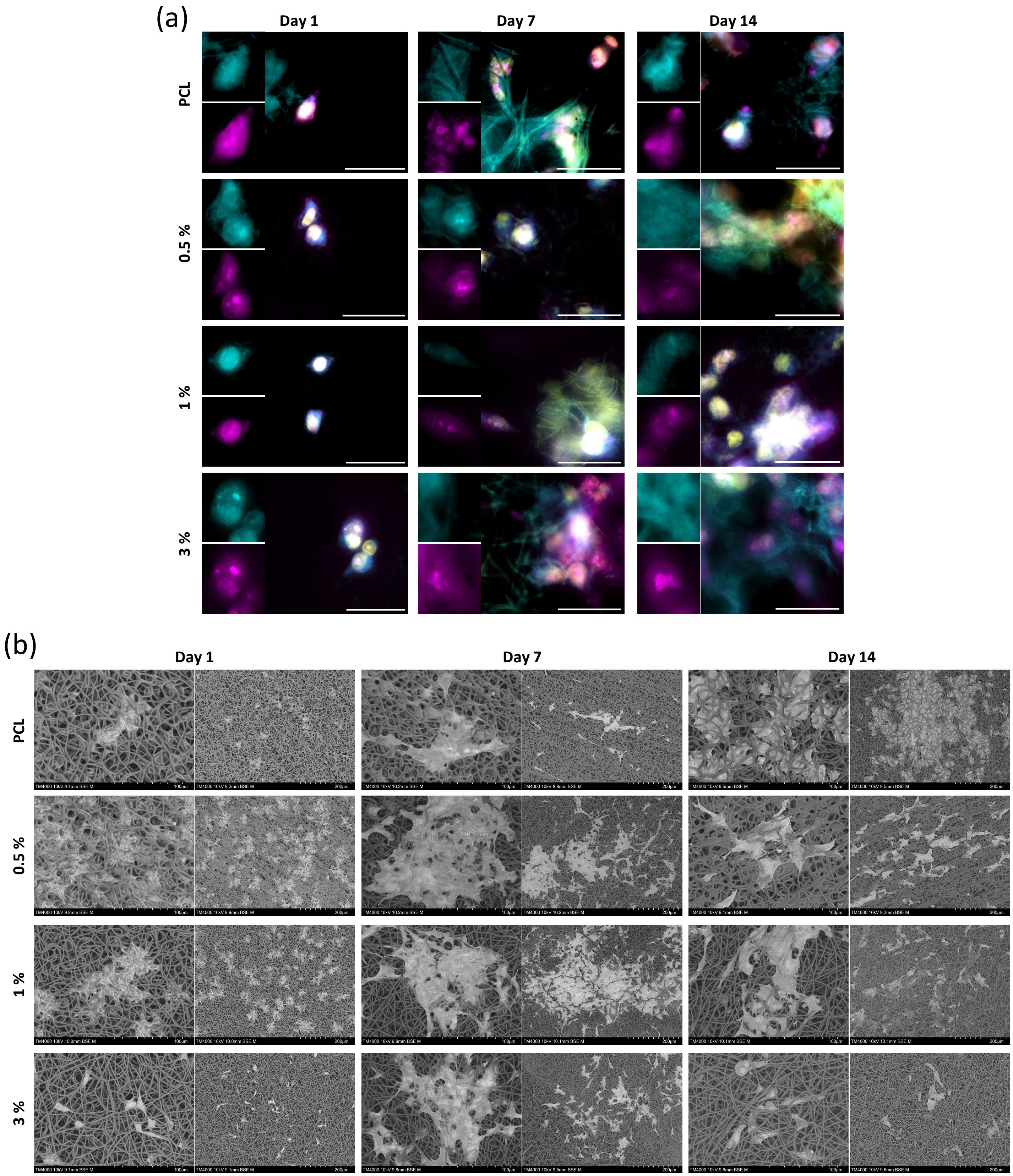

Supplementary Figure 19. – (a) Images of electrospun PCL-only and 0.5, 1 and 3% adenosine scaffolds seeded with Nthy-ori 3-1 cells and immunofluorescently stained for thyroglobulin (Tg, cyan), Ki67 (magenta) and DAPI (yellow) at days 1, 7 and 14 of culture. Scale bars = 40µm, x60 magnification. (b) SEM images of electrospun PCL-only and 0.5, 1 and 3% adenosine scaffolds seeded with Nthy-ori 3-1 cells and stained with osmium at day 1, 7 and 14 of culture. Left columns: scale bar=100µm, x1500 magnification. Right columns: scale bar=200µm, x500 magnification.
